# Micropeptide SCAPEP triggers lung adenocarcinoma tumorigenesis via regulating autophagy by promoting CDK15-mediated phosphorylation of vimentin

**DOI:** 10.1038/s41419-026-08767-1

**Published:** 2026-05-06

**Authors:** Xuefei Shi, Qiuhui Li, Fengqi Nie, Xuting Xu, Yili Shen, Hui Shen, Kai Chen, Bin Wang, Kaihua Lu, Shunli Dong, Liu Xianghua

**Affiliations:** 1 Department of Respiratory Medicine, Huzhou Central Hospital, Fifth School of Clinical Medicine of Zhejiang Chinese Medical University, Huzhou, PR China; 2 Huzhou Key Laboratory of Precision Diagnosis and Treatment in Respiratory Diseases, Huzhou University, Huzhou, PR China; 3 Department of Biochemistry and Molecular Biology, School of Basic Medical Sciences, Nanjing Medical University, Nanjing, PR China; 4 Department of Oncology, Second Affiliated Hospital, Nanjing Medical University, Nanjing, PR China; 5 Department of Central Laboratory, Huzhou Central Hospital, Affiliated Central Hospital, Huzhou University, Huzhou, PR China; 6 Department of Oncology, First Affiliated Hospital, Nanjing Medical University, Nanjing, PR China; 7 Department of Oncology, The Affiliated Jiangning Hospital of Nanjing Medical University, Nanjing, PR China

**Keywords:** Oncogenes, Non-small-cell lung cancer

## Abstract

Lung adenocarcinoma is one of the most common forms of lung cancer with a low five-year survival rate. The roles of the novel proteins and peptides encoded by circular RNAs (circRNAs) in cancer are emerging. However, the functions and underlying molecular mechanisms of the peptides that affect lung adenocarcinoma progression are yet to be elucidated. In this study, we characterized a lung-adenocarcinoma-associated small peptide (SCAPEP) encoded by circRNA_0065214 via the IRES element. Tumors with a large diameter, lymphatic metastasis, or advanced stage have high SCAPEP levels. Furthermore, modulation of SCAPEP expression can regulate cell proliferation, metastasis, and autophagy in vitro or in vivo. We found that the up-regulation of methionine synthase reductase (MTRR) by regulating the phosphorylation of vimentin Ser56 in SCAPEP is a key mechanism driving the aggressiveness of SCAPEP -high lung adenocarcinoma. Consistent with these findings, MTRR overexpression reversed the effects of SCAPEP depletion. Altogether, our observations provide novel insights into how oncogenic peptides crosstalk with autophagy and contribute to lung adenocarcinoma tumorigenesis.

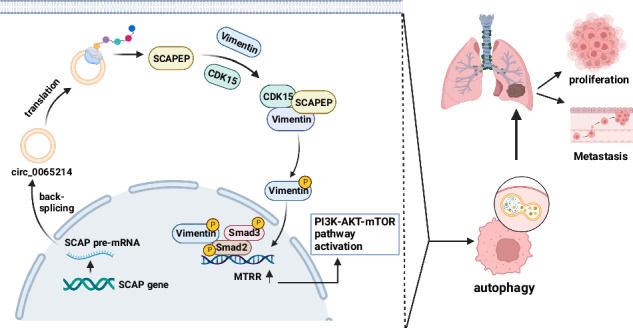

## Introduction

Lung cancer is a devastating disease and remains the primary cause of cancer-related deaths worldwide [1]. Lung adenocarcinoma (LUAD) and squamous carcinoma (LUSC) are the two main pathological types of lung cancer and account for ~40% of all newly diagnosed lung cancer cases [2]. Although recent advances in molecular targeted therapy and immunotherapy have remarkably improved the outcome of patients with lung adenocarcinoma, a large proportion of patients do not benefit from these treatments owing to drug resistance and insensitivity to PD1/PD-L1 inhibitors [3, 4]. As a result, the 5-year survival rate of patients with lung adenocarcinoma remains at approximately 20%, which remains unsatisfactory [5]. Therefore, an accurate understanding of the molecular pathways that underlie lung adenocarcinoma carcinogenesis and progression is required.

In recent years, emerging evidence has demonstrated that circular RNAs (circRNAs) contain numerous uncharacterized open reading frames that yield many unannotated proteins or peptides driven by the IRES element or N6-methyladenosines (m^6^A) modification, which broadens our understanding of molecular biology [6, 7]. These proteins and peptides contribute to the pathogenesis and progression of cancers and other diseases by regulating cell differentiation, proliferation, metabolism, and immune evasion [8–10]. For example, in GBM cells, HEATR5B, encoded by circHEATR5B, suppresses aerobic glycolysis and proliferation by reducing the phosphorylation of JMJD5 [11]. Similarly, EZH2-92aa mediates the immune evasion of GBM stem cells from natural killer cells via the inhibition of surface NKG2D ligands [12]. Nevertheless, the presence of these circRNA-derived proteins or peptides and their functions in lung cancer remain largely elusive. Parsing their biological functions and underlying mechanisms may provide valuable clinical insights.

Autophagy is an evolutionarily conserved catabolic degradation process responsible for the recycling of damaged organelles and the degradation of intracellular substances, and it is important for the maintenance of energy balance and cellular homeostasis during development or under different stress conditions [13, 14]. This “self-eating” process is mediated by lysosomal and autophagy-related proteins, including LC3A, LC3B, and LC3C [15]. Cellular autophagic activity is highly conserved and tightly regulated in eukaryotes. Dysregulation of autophagy results in lipid accumulation, damage, and inflammation, leading to the onset and development of multiple diseases, including cardiovascular, metabolic, and neoplastic diseases [16, 17]. To date, the dual role of autophagy in the different stages of lung cancer progression remains controversial. For instance, there is evidence that inactivation of the autophagy gene ATG5 plays a role in promoting KRAS-driven non-small cell lung cancer (LUAD) at an early stage; however, autophagy is necessary for adenocarcinoma progression at a later stage [18]. Recently, Miao et al. demonstrated that LC3A-mediated autophagy crosstalk with SOX2 proliferation signaling determines the plasticity of lung cancer cells [19]. Therefore, it is essential to understand the fine-grained molecular regulation of autophagy to better understand its role in lung adenocarcinoma development.

Here, we sought to reveal the translated circRNA profile in lung adenocarcinoma using high-throughput circRNA microarray analysis and ribosome-nascent chain complex sequencing (RNC-seq) of lung adenocarcinoma and paired normal lung tissue samples. We characterized a lung-adenocarcinoma-associated small peptide derived from circ_0065214 (named SCAPEP, 56 amino acids [aa] in length) through comprehensive analysis and point mutation assays. Moreover, we investigated the function and corresponding mechanism of action of SCAPEP in lung adenocarcinoma cells and assessed its clinical significance in patients with lung adenocarcinoma.

## Result

### Characterization of highly expressed CircRNA with translational potential in LUAD

**Fig. 1:**
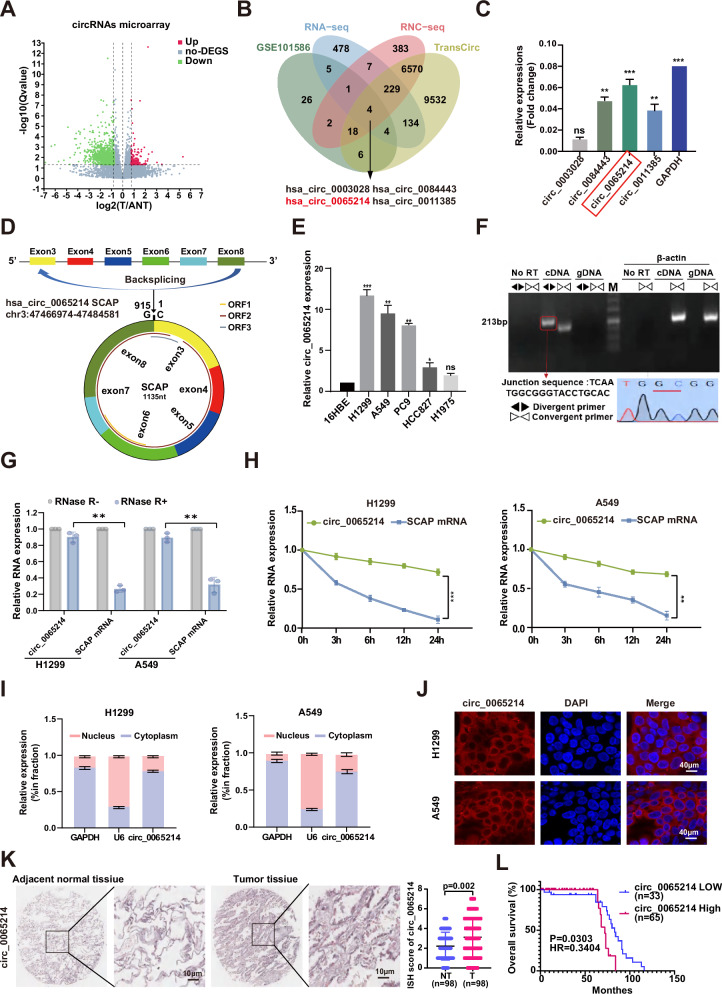
Through comprehensive analysis of different sequencing datasets, including RNA-seq, RNC-seq, and Ribo-seq, circ_0065214 with potential encoding capability was identified in LUAD cells. **A** The figure illustrates differentially expressed circRNAs detected by RNA-seq in five pairs of human lung adenocarcinoma tissues and matched nontumor tissues. **B** The Venn diagram depicts four differentially expressed circRNAs with encoding potential identified in LUAD, based on a cross-matching analysis of differentially expressed circRNAs from RNA-seq, ribosome-newly synthesized peptide complex sequencing (RNC-seq), and polysome profiling analysis (Ribo-seq) in five pairs of lung adenocarcinoma tissues, as well as differential expression circRNAs from LUAD circRNAs microarray (GSE1015886) data obtained from the GEO dataset. **C** The expression levels of four circRNAs with encoding potential in LUAD tissues were analyzed using qRT-PCR. **D** circ_0065214 is back-spliced by exons 3 and 8 of SCAP, and it contains three ORFs. **E** qRT-PCR was employed to examine the expression levels of circ_0065214 in various LUAD cell lines and normal lung adenocarcinoma tissues. **F** The divergent primers detected circ_0065214 in cDNA but not in gDNA. β-actin was used as a control for a linear RNA transcript. **G** qRT-PCR analysis of circ_0065214 and SCAP mRNA after treatment with RNase R in LUAD cells, revealing a significant resistance of circ_0065214 to RNase R. **H** The relative RNA levels of circ_0065214 and SCAP were determined at different time points in LUAD cells treated with Actinomycin D. **I** The sub-cellular distribution of circ_0065214 was mostly present in the cytoplasm by the nuclear mass separation assay. **J** The location of circ_0065214 in LUAD cells was identified by FISH with junction-specific probes specific to circ_0065214. scale bar: 40 μm. **K** The level of circ_0065214 was analyzed by in situ hybridization on LUAD tissue microarray, showing that circ_0065214 was up-regulated in LUAD tissues compared with normal tissues. scale bar: 10 μm. **L** Overall survival in patients with high and low circ_0065214 levels in LUAD tissue samples. Data are represented as mean ± SD. Differences between the groups were evaluated using Student’s *t* test, ns indicates no significance, *P < 0.05; **P < 0.01; ***P < 0.001.

Increasing evidence has demonstrated the ability of circRNAs to encode micropeptides or microproteins that contribute to tumor development [20]. However, the knowledge of circRNAs with coding potential in LUAD remains limited. To investigate circRNAs that exhibit significant coding potential in LUAD, we first used a circRNA microarray to identify differentially expressed circRNAs in five pairs of LUAD tissues and their corresponding adjacent normal tissues, resulting in the identification of 1489 significantly differentially expressed circRNAs (Fig. 1A). Additionally, we downloaded circRNA microarray data (GSE101586) from the Gene Expression Omnibus database, which included the same five pairs of lung adenocarcinoma tissues. Furthermore, we conducted RNC-seq analysis on LUAD cell lines to identify circRNAs that interact with ribosomes and searched the TransCirc database, which contains predicted circRNAs with coding potential and their encoded functional peptides. Through the intersection of differentially expressed circRNAs from these four datasets, we successfully identified four circRNAs that emerged as potential candidates (Fig. 1B). Out of these four candidate circRNAs, our attention was primarily directed towards circRNAs that demonstrated amplified differential expression between the cancerous and normal groups. Quantitative real-time PCR (qRT-PCR) analysis revealed that hsa_circ_0065214 exhibited significantly higher expression levels in LUAD tissues than the other three candidate circRNAs (Fig. 1C). Based on these findings, hsa_circ_0065214 was selected as the primary target for further investigation in the next study.

Hsa_circ_0065214 is generated through the back-splicing of exons 3-8 from the linear transcription of the host gene SCAP, resulting in an RNA molecule comprising 915 nucleotides in length, also known as circSCAP, which indicates that it possesses three potential open reading frames (ORFs) (Fig. 1D), further suggesting its potential for encoding proteins. We first employed qRT-PCR to evaluate the expression level of circ_0065214 in different LUAD cells and found that circ_0065214 was highly expressed in LUAD cells compared to normal lung epithelial cells, with particularly high expression levels in H1299 and A549 cells (Fig. 1E). Consequently, we identified circ_0065214 in these two LUAD cell lines. Subsequently, PCR results demonstrated that circ_0065214 could be amplified using divergent primers from the cDNA of LUAD cells but not from genomic DNA, whereas the linear transcript of the actin gene could be amplified using convergent primers from both sources. Sequencing verified the presence of a back-splicing site in the PCR product, which was amplified using divergent primers (Fig. 1F). Additionally, the RNase R degradation assay revealed degradation of the linear transcript of SCAP upon treatment with RNase R, whereas circ_0065214 exhibited resistance to this treatment (Fig. 1G). Furthermore, circ_0065214 displayed greater stability than SCAP after actinomycin D treatment (Fig. 1H). Cellular component extraction and RNA-FISH showed that circ_0065214 was mainly localized in the cytoplasm of LUAD cells (Fig. 1I, J). To better understand the role of circ_0065214 in LUAD, we determined circ_0065214 levels in 90 tissue microarrays using in situ hybridization. The results showed that circ_0065214 was up-regulated in LUAD tissues, and Kaplan-Meier analysis revealed that LUAD patients with low circ_0065214 had superior overall survival versus those with high circ_0065214 expression (Fig. 1K, L). In summary, these findings strongly suggest that circ_0065214 is a stable circular transcript with coding potential in LUAD cells.

### circ_0065214 encodes a novel micropeptide, SCAPEP

**Fig. 2:**
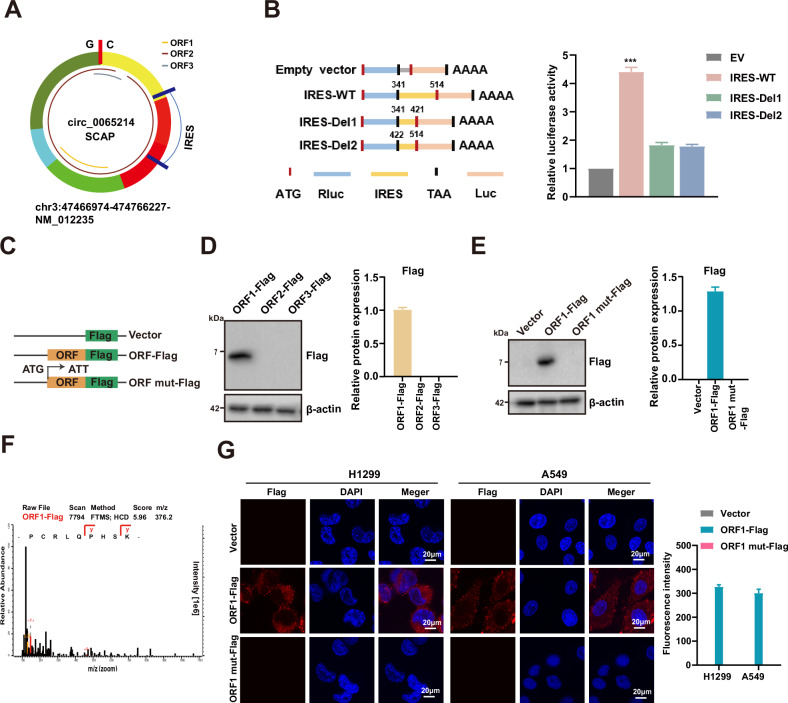
circ_0065214 encodes a small uncharted protein SCAPEP. **A** Illustrate the IRES sequence of circ_0065214 along with its three contained ORFs. **B** Insert the IRES sequence of circ_0065214 or its different truncations between the Rluc and Luc reporter genes, which have independent start (AUG) and stop codons (UGA). The schematic structure of these vectors was shown (left). The luciferase activity ratio of Luc/Rluc was assessed in the aforementioned vectors (right). **C** Two distinct plasmids were constructed. ORF-Flag: The sequences of the three ORFs of circ_0065214 were cloned into the Plvx-flag expression vector individually ORF mut-flag: ORF sequence with start codon mutant (ATG → ATT) was cloned into the Plvx-flag expression vector. **D**, **E** The expression level of flag-label ORFs and ORF mut was detected by western blot analysis. **F** The lysate of cells transfected with ORF1-Flag was separated by SDS-PAGE, and protein bands around the expected molecular weight were excised. Mass spectrometry analysis was performed to identify specific peptide segments derived from the translation of ORF1-Flag protein. **G** ORF1-Flag and ORF mut-Flag were transfected into LUAD cells, and their expression and localization in the cells were examined using immunofluorescence with Flag antibody, with the empty vector as a control. scale bars, 20 μm. Data are represented as mean ± SD. Differences between the groups were evaluated using Student’s *t* test, *P < 0.05, **P < 0.01, ***P < 0.001.

To explore the encoding potential of circ_0065214, we utilized circRNADb software for analysis, which revealed the presence of an internal ribosome entry site (IRES) and ORFs in circ_0065214 (Fig. 2A). We first used a luciferase assay to detect the IRES activity of circ_0065214. Full-length or truncated IRES sequences were cloned into the pGL3-Basic vector, and the predicted IRES activity was evaluated using a dual-luciferase assay, with the empty pGL3-Basic vector as a negative control. The results showed that the full-length IRES sequence exhibited higher luciferase activity, indicating a stronger capability for protein translation initiation, whereas the truncated vector had no significant effect (Fig. 2B). The ORF is considered the structural element driving the coding potential of a gene [21, 22]. To validate our hypothesis that circ_0065214 is translatable, we constructed a series of expression plasmids for the three putative ORFs. The ORF sequences were modified by adding a 3xFlag tag before the stop codon and were cloned separately into synthetic circRNA expression vectors with flanking sequences (Fig. 2C). If the circ_0065214 sequence was successfully circularized and its ORF was capable of translation, a flag tag was detected. Western blot analysis demonstrated successful translation of the second ORF of circ_0065214, resulting in the production of a micropeptide (Fig. 2D). To confirm the ability of ORF1 to encode a novel micropeptide, we engineered an expression plasmid with a mutation in the start codon of ORF1. Western blotting further confirmed that ORF1 could be translated, and it produced a 56-aa micropeptide (Fig. 2E). The presence of specific peptide segments in this micropeptide was also detected using mass spectrometry (Fig. 2F and Table S6). Meanwhile, immunofluorescence results also showed that red fluorescence could be detected only in LUAD cells transfected with ORF1-Flag, while it could not be detected successfully after initiation codon mutation (Fig. 2G). These findings underscore the encoding potential of circ_0065214, which was assigned the name SCAPEP and represents a protein encoded by circSCAP (circ_0065214).

### Increased levels of SCAPEP in LUAD are positively correlated with poor prognosis in patients

**Fig. 3:**
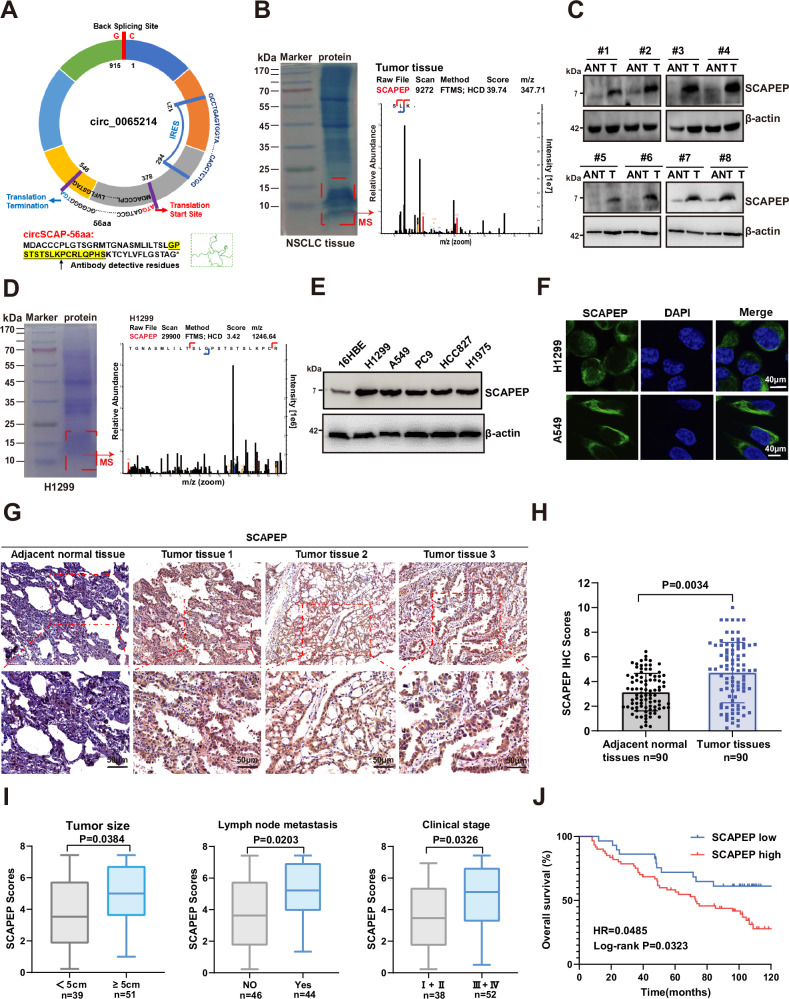
SCAPEP exhibits endogenous and natural expression, while its upregulation in cancers is associated with the prognosis of LUAD patients. **A** The diagram depicts circ_0065214 containing translatable ORF and IRES sequences, with the specific amino acid sequences recognized by the custom antibody targeting the micropeptide SCAPEP, translated from ORF1, highlighted in yellow. **B** Protein samples from LUAD tissues were subjected to SDS-PAGE separation, followed by gel excision between 15 to 5 kDa for mass spectrometry analysis. **C** Western blot analysis of SCAPEP expression in 10 cases of LUAD and paired lung tissues. **D** Protein samples from LUAD cells were subjected to SDS-PAGE separation, followed by gel excision between 25 to 10 kDa for mass spectrometry analysis. **E** Expression of SCAPEP in different LUAD cell lines analyzed by western blot. **F** Immunofluorescence staining to demonstrate the subcellular localization of SCAPEP in LUAD cells. scale bars, 40 μm. **G** Representative images of immunohistochemical staining of SCAPEP in tissue microarrays containing 90 cases of LUAD and adjacent normal tissues. scale bars, 50 μm. **H** Differences in immunohistochemical scoring of SCAPEP between adjacent normal tissues and tumor tissues are represented by a box plot. **I** Correlation analysis of SCAPEP expression levels with tumor size, clinical stage and lymph node metastasis in 90 LUAD patients. **J** LUAD patients exhibiting elevated levels of SCAPEP demonstrated decreased overall survival compared to individuals with lower SCAPEP levels.

circ_0065214 is derived from the parental SCAP gene (Fig. 3A), and we hypothesized that SCAPEP originates from the SCAP protein. Amino acid sequence alignment revealed that the micropeptide SCAPEP, encoded by circ_0065214, does not share homology with the SCAP protein. To further exclude the possibility that this micropeptide originated from linear SCAP mRNA, we subjected the gel slices near 7 kDa to SDS-PAGE and mass spectrometry analyses to explore the endogenous expression of SCAPEP in LUAD cells and tissues (Fig. 3B, D). The results revealed successful detection of SCAPEP in LUAD cells and tissues, indicating its natural and endogenous expression. Subsequently, we generated a polyclonal antibody, anti-SCAPEP, targeting the specific peptide segments of SCAPEP. Western blot analysis was used to assess SCAPEP expression in LUAD cells and tissues. Furthermore, the presence of endogenous SCAPEP was confirmed in fresh primary cancer tissues and their corresponding adjacent non-tumor tissues, and SCAPEP was found to be highly expressed in LUAD tumor tissues compared to adjacent normal lung tissues (Fig. 3C). In addition, SCAPEP levels were upregulated in LUAD cells compared to normal lung epithelial cells (Fig. 3E). Meanwhile, we discovered that natural endogenous SCAPEP was present in LUAD cells and was primarily localized in the cytoplasm (Fig. 3F). These findings suggested that high SCAPEP expression levels in LUAD cells may play an oncogenic role. To further investigate the clinical relevance of aberrantly high SCAPEP expression levels in LUAD, we used immunohistochemistry to detect the expression of SCAPEP in 90 pairs of lung cancer tissues and their adjacent normal tissues and analyzed the follow-up survival data. The results revealed a significant upregulation of SCAPEP expression in lung cancer tissues compared to adjacent normal tissues (Fig. 3G, H), and its increased expression levels were positively associated with a larger tumor size, lymph node metastasis, and advanced clinical stage (Fig. 3I and Supplementary Table S1). Kaplan–Meier analysis demonstrated that patients with higher SCAPEP expression levels in tumors had a poorer prognosis (Fig. 3J). Collectively, this data provided evidence to suggest that SCAPEP is endogenously and naturally synthesized within human LUAD cells and tissues.

### SCAPEP promotes the proliferation and metastasis of LUAD cells both in vitro and in vivo

Having confirmed the overexpression of SCAPEP in LUAD cells and tissues, our subsequent efforts were directed towards exploring the functional implications of SCAPEP in the progression of LUAD. We designed two short hairpin RNAs (shRNAs) targeting the circ_0065214 junction site sequence and employed lentiviral packaging to generate a stable SCAPEP-knockdown cell line. As shown in Supplementary Fig. S1A, B, qRT-PCR results showed that the expression of circ_0065214 was successfully knocked out by the two shRNAs, but the mRNA of linear SCAP could not be knocked out in H1299 or A549 cells. Western blot analysis demonstrated that these two shRNAs effectively downregulated the expression of SCAPEP without affecting the expression of full-length SCAP (Supplementary Fig. S1C). Subsequently, we conducted a series of functional cellular experiments to explore the role of SCAPEP in LUAD progression. Results from the CCK-8 and colony-formation assays indicated that the proliferation of LUAD cells stably transfected with circ_0065214 shRNAs was significantly impaired compared to the control group (Supplementary Fig. S1D, E). Likewise, the transwell assay results demonstrated that downregulation of circ_0065214 led to weakened migration and invasion abilities of LUAD cells (Supplementary Fig. S1F, G). We conducted the aforementioned cell function experiments in LUAD cells stably overexpressing SCAPEP. The results revealed that compared to the control group, overexpression of SCAPEP significantly enhanced the proliferation and migration of LUAD cells (Supplementary Fig. S1H–L). These findings suggest that SCAPEP promotes the development of LUAD.

**Fig. 4:**
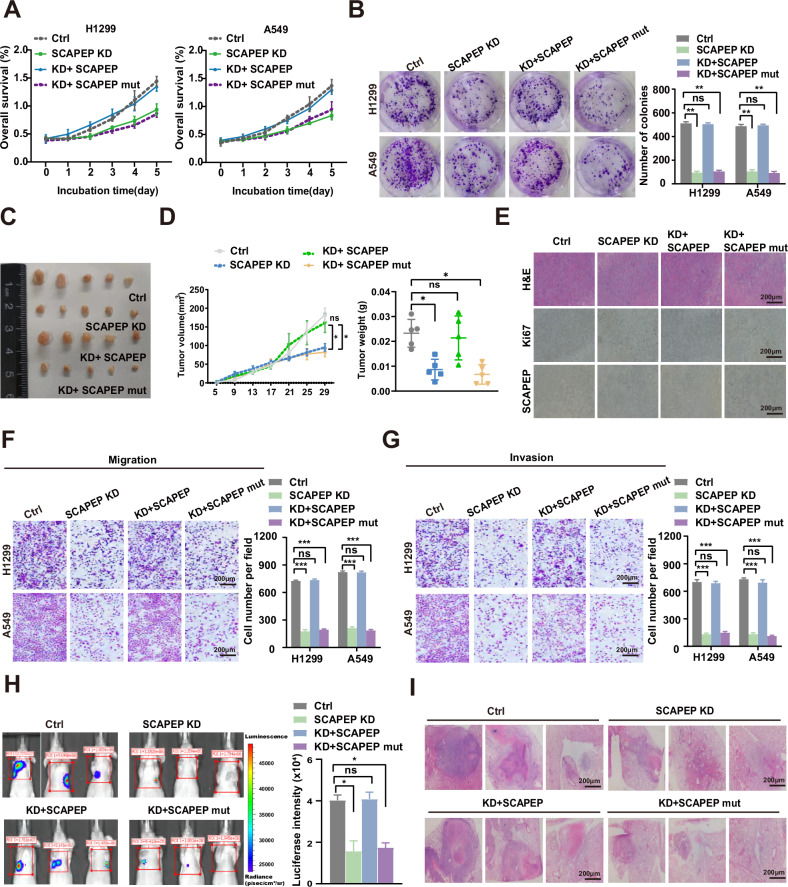
SCAPEP promotes the growth and metastasis of LUAD both in vitro and in vivo. LUAD cells with stable knockdown of SCAPEP were re-expressed with SCAPEP, SCAPEP mut, establishing four groups of LUAD cells: Ctrl, SCAPEP KD, KD + SCAPEP, and KD + SCAPEP mut, to evaluate the proliferative capacity of LUAD cells using CCK-8 assay (**A**) and colony formation experiments (**B**). **C** Subcutaneous injection of the aforementioned cells into nude mice resulted in xenograft tumor growth (*n* = 5), as demonstrated in representative images of the experimental model. **D** The tumor weight and volume of the corresponding xenograft tumors were recorded and statistically analyzed, respectively. **E** Subcutaneous tumor samples were collected for H&E staining and immunohistochemical analysis to assess the expression levels of SCAPEP and Ki-67. scale bar: 200 μm. **F**–**G** Transwell assay was performed to evaluate the migratory and invasive capabilities of the four groups of LUAD cells mentioned above. scale bar: 200 μm. **H** The four groups of LUAD cells were labeled with luciferase and injected via the tail vein into nude mice to establish a lung metastasis model (2 × 10^6^ cells/mouse) Bioluminescence imaging was performed to observe the luciferase activity in the lungs of mice (*n* = 3). **I** H&E staining assesses the quantity and size of nodules formed in the lung metastasis model. scale bar: 200 μm. Data are represented as mean ± SD. Differences between the groups were evaluated using Student’s *t* test, ns indicates no significance, *P < 0.05, **P < 0.01, ***P < 0.001.

Because of the utilization of shRNAs for SCAPEP knockdown as described above, circ_0065214 was also knocked down. To determine the biological effects of SCAPEP independent of circ_0065214, we transfected a SCAPEP-overexpression plasmid or a SCAPEP mutant plasmid (with a mutation in the start codon) into LUAD cells with SCAPEP stably knocked down, and established the following four sets of LUAD cell lines: Ctrl, SCAPEP KD, KD + SCAPEP, and KD + SCAPEP mut. Western blotting was performed to detect the efficiency of SCAPEP expression knockdown in the indicated LUAD cells. As shown in Fig. 4A, B, the results of CCK-8 and colony formation experiments demonstrated that knockdown of SCAPEP suppressed the proliferative capacity of LUAD cells, whereas re-expression of SCAPEP restored their proliferative ability. At the same time, western blotting detection of proliferation marker PCNA also obtained consistent results (Supplementary Fig. S2A). However, the re-expression of SCAPEP mut did not yield similar outcomes. Using the aforementioned four groups of LUAD cells, we established a subcutaneous xenograft tumor model in nude mice. Consistent with the aforementioned findings, SCAPEP knockdown impaired tumor growth, whereas the reintroduction of SCAPEP, but not the SCAPEP mutant, into SCAPEP-knockdown LUAD cells restored tumor growth (Fig. 4C). A consistent phenomenon was also observed for tumor volume/weight, and for SCAPEP and Ki67 expression levels in tumor tissues from xenograft nude mice (Fig. 4D, E). To investigate the effect of SCAPEP on the metastatic potential of LUAD cells, transwell experiments and transfer-related protein indexes were conducted using the aforementioned four cell groups, and a lung metastasis model was established in nude mice. The results demonstrated that SCAPEP knockdown significantly inhibited the metastatic ability of LUAD cells both in vitro and in vivo. However, the metastatic capability of LUAD cells was restored upon re-expression of SCAPEP, but not of SCAPEP mut (Fig. 4F–I and Supplementary Fig. S2B). Collectively, these results showed that SCAPEP acts as an oncogenic protein that promotes the proliferation and metastasis of LUAD.

### SCAPEP modulates the phosphorylation of vimentin in LUAD cells

**Fig. 5:**
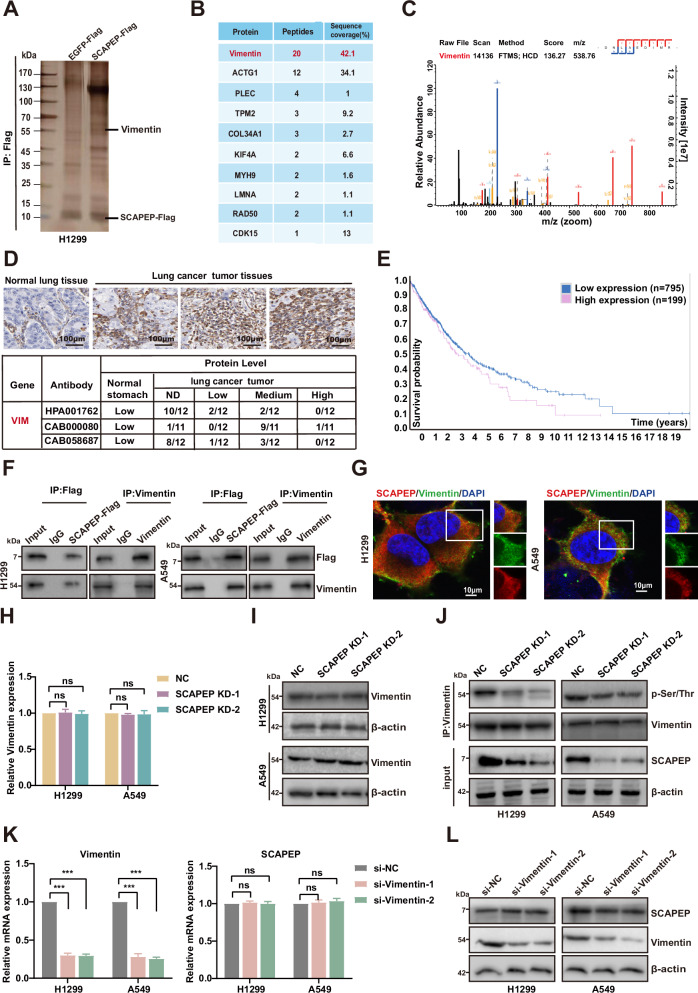
SCAPEP modulates the phosphorylation of vimentin in LUAD cells. **A** Proteins interacted with the SCAPEP were identified by interactomics analysis. **B** Mass spectrometry identified the top ten potential proteins interacting with SCAPEP. Table S5 for detailed mass spectrometry information. **C** Present the vimentin-specific amino acid sequence identified by mass spectrometry. **D** Analyze the expression of vimentin in LUAD tumor and adjacent normal tissue specimens using IHC data obtained from the HPA. **E** Perform Kaplan-Meier Plotter analysis to assess the correlation between vimentin expression levels and overall survival of LUAD patients. **F** Evaluate the binding potential of SCAPEP with vimentin using a co-IP assay. **G** Immunofluorescence analysis to assess the subcellular localization of SCAPEP and vimentin in LUAD cells. scale bar: 10 μm. **H**, **I** Perform qRT-PCR and western blot analysis to determine the RNA and protein expression levels of vimentin in LUAD cells with SCAPEP knockdown. **J** SCAPEP knocking down led to the reduced phosphorylation level of vimentin in LUAD cells. **K**, **L** The RNA and protein expression levels of vimentin and SCAPEP were determined by qRT-PCR and western blot in LUAD cells with vimentin knockdown. Data are represented as mean ± SD. Differences between the groups were evaluated using Student’s *t* test, ns indicates no significance, ***P < 0.001.

To elucidate the molecular mechanism of SCAPEP in promoting LUAD progression, stable LUAD cells expressing SCAPEP-Flag were employed for co-immunoprecipitation (co-IP) to enrich proteins interacting with SCAPEP. An empty EGFP-Flag empty vector was used as a control, and silver staining was used to detect enriched proteins in both groups (Fig. 5A). A total of 50 proteins interacting with SCAPEP were identified through mass spectrometry, and these proteins were ranked based on the number of specific peptide segments and sequence coverage (%), with vimentin being the top-ranked protein (Fig. 5B, C). Vimentin, a prominent member of the intermediate filament protein family, is widely expressed in normal mesenchymal cells and plays a crucial role in maintaining cellular integrity and imparting resistance against stress [23, 24]. Enhanced vimentin expression has been documented in different epithelial cancers such as lung cancer, breast cancer, prostate cancer, gastrointestinal tumors, central nervous system tumors, and other malignancies [24–29]. Vimentin overexpression in cancer is strongly associated with accelerated tumor growth, invasiveness, and a poor prognosis [30]. Using immunohistochemical data from the HPA database, we analyzed the immunohistochemical results of vimentin in 35 LUAD tissue samples, and identified a downregulation of vimentin expression in normal lung tissue, while it was significantly upregulated in LUAD tissue (Fig. 5D). Simultaneously, the Kaplan-Meier Plotter analysis of the progression-free survival in 994 LUAD patients revealed that patients with high vimentin expression had a poorer prognosis, suggesting that vimentin may serve as a molecular biomarker for the prognosis of LUAD (Fig. 5E). We further validated the interaction between SCAPEP and vimentin in LUAD using co-IP and demonstrated their co-localization in the cytoplasm using immunofluorescence (Fig. 5F, G). Interestingly, we observed that SCAPEP knockdown did not affect the RNA and protein levels of vimentin; however, it significantly impacted the phosphorylation levels of vimentin (Fig. 5H–J). Conversely, knockdown of vimentin did not affect the RNA and protein levels of SCAPEP (Fig. 5K, L). These results suggest that SCAPEP may influence the phosphorylation modification of vimentin at the post-translational level.

### SCAPEP regulates vimentin phosphorylation by promoting CDK15 binding to vimentin

**Fig. 6:**
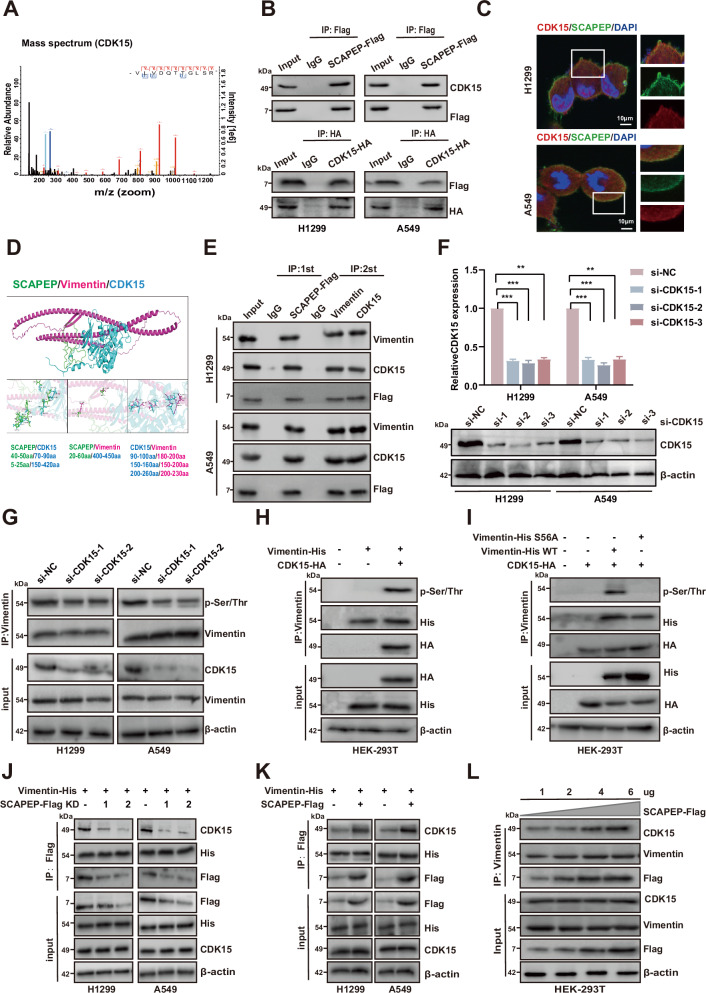
SCAPEP regulates vimentin phosphorylation by promoting CDK15 binding to vimentin. **A** Mass spectrometry identified the CDK15-specific amino acid sequence in the protein sample enriched by co-IP, which interacts with SCAPEP. **B** Co-IP experiments revealed the mutual interaction between SCAPEP and CDK15 in LUAD cells. **C** Immunofluorescence analysis demonstrated colocalization of SCAPEP and CDK15 in LUAD cells. scale bar: 10 μm. **D** The AlphaFold software predicts the ternary complex binding mode diagram of SCAPEP, vimentin and CDK15. **E** The consecutive Co-IP assay was performed to detect the mutual binding of SCAPEP/vimentin/CDK15. **F** The RNA and protein expression levels of CDK15 were determined by qRT-PCR and western blot in LUAD cells with CDK15 knockdown. **G**, CDK15 knocking down led to the reduced phosphorylation level of vimentin in LUAD cells. **H** Overexpression of CDK15 enhances the phosphorylation levels of vimentin in HEK-293T cell. **I** Co-IP assay was performed to detect the phosphorylation levels of vimentin after co-transfection of CDK15 and vimentin WT or vimentin mut (S56A) in HEK-293T cells. **J** Co-IP assay was conducted to assess the binding affinity between vimentin and CDK15 after SCAPEP knockout. **K** IP and western blot assays indicating the interaction of vimentin and CDK15 in SCAPEP overexpressing LUAD cells. **L** SCAPEP-Flag was co-transfected with vimentin and CDK15 in a dose-dependent manner into HEK-293T cells, followed by a co-IP assay to detect the interaction between vimentin and CDK15.

Phosphorylation is the most widespread covalent modification of proteins in post-translational modification. In eukaryotes, reversible phosphorylation of specific serine, threonine, and tyrosine residues in proteins controls essential functions ranging from metabolism and gene expression to cell cycle regulation and cellular stress response [31, 32]. Phosphatases, together with protein kinases, regulate the phosphorylation state of proteins in cells [33, 34]. Previous mass spectrometry identification of proteins interacting with SCAPEP revealed the presence of CDK15 (Fig. 6A and Supplementary Table S5), a member of the cyclin-dependent kinase family, and we have already demonstrated that SCAPEP can regulate the phosphorylation levels of vimentin. Therefore, we hypothesize that SCAPEP may influence the phosphorylation levels of vimentin through CDK15. Firstly, we confirmed the interaction between CDK15 and SCAPEP through Co-IP, and observed the colocalization of both proteins in the cytoplasm (Fig. 6B, C). Subsequently, utilizing AlphaFold prediction software, we identified the potential for SCAPEP, vimentin and CDK15 to form a trimeric complex (Fig. 6D). Further validation of this complex formation was achieved through sequential immunoprecipitation assays, confirming the formation of a complex involving SCAPEP, vimentin and CDK15 (Fig. 6E). Next, we further investigated whether vimentin serves as a substrate for CDK15. We successfully knocked down the RNA and protein expression levels of CDK15 in LUAD cells using siRNAs interference sequences (Fig. 6F). Concurrently, we observed a significant decrease in the phosphorylation levels of vimentin following CDK15 knockdown (Fig. 6G). Conversely, upon overexpression of CDK15, the phosphorylation levels of vimentin were upregulated (Fig. 6H). To determine whether vimentin is a substrate of CDK15, we performed an in vitro kinase assay, which showed a dose-dependent increase in vimentin serine/threonine phosphorylation (p-Ser/Thr) upon CDK15 treatment, indicating that CDK15 directly phosphorylates vimentin (Supplementary Fig. S3A, B). In addition, we utilized the PhosphoSitePlus database to further determine the potential residues of vimentin phosphorylated by CDK15. We found that two other members of the cyclin-dependent kinase family, CDK1 and CDK5, are capable of promoting phosphorylation modification at the Ser56 site of vimentin [35, 36]. Therefore, we further validated whether CDK15, as a member of the same family, could also potentially influence the phosphorylation modification of vimentin at the Ser56 site. We constructed a plasmid, vimentin S56A, with a mutation at the Ser56 site of vimentin, and co-transfected it along with vimentin-His WT and CDK15-HA into HEK293T cells. Co-IP results indicated that the mutation at the Ser56 site significantly affected the phosphorylation modification of vimentin (Fig. 6I). To further pinpoint the potential residue of vimentin phosphorylated by CDK15, we conducted in vitro kinase assays, which confirmed Serine 56 (S56) as a likely phosphorylation site and furthermore revealed that SCAPEP enhances CDK15-mediated phosphorylation at this specific site (Supplementary Fig. S3C, D). Furthermore, we observed that stable knockdown of SCAPEP in LUAD cells attenuated the interaction between Vimentin and CDK15, while overexpression of SCAPEP enhanced their interaction (Fig. 6J, K). Meanwhile, we found that with the sustained increase in SCAPEP abundance, the interaction between vimentin and CDK15 showed a dose-dependent enhancement (Fig. 6L). In summary, these results suggest that SCAPEP regulates the phosphorylation modification of vimentin by promoting its binding with CDK15 in LUAD cells.

### SCAPEP promotes autophagy occurrence in LUAD cells through co-regulating MTRR expression with vimentin

**Fig. 7:**
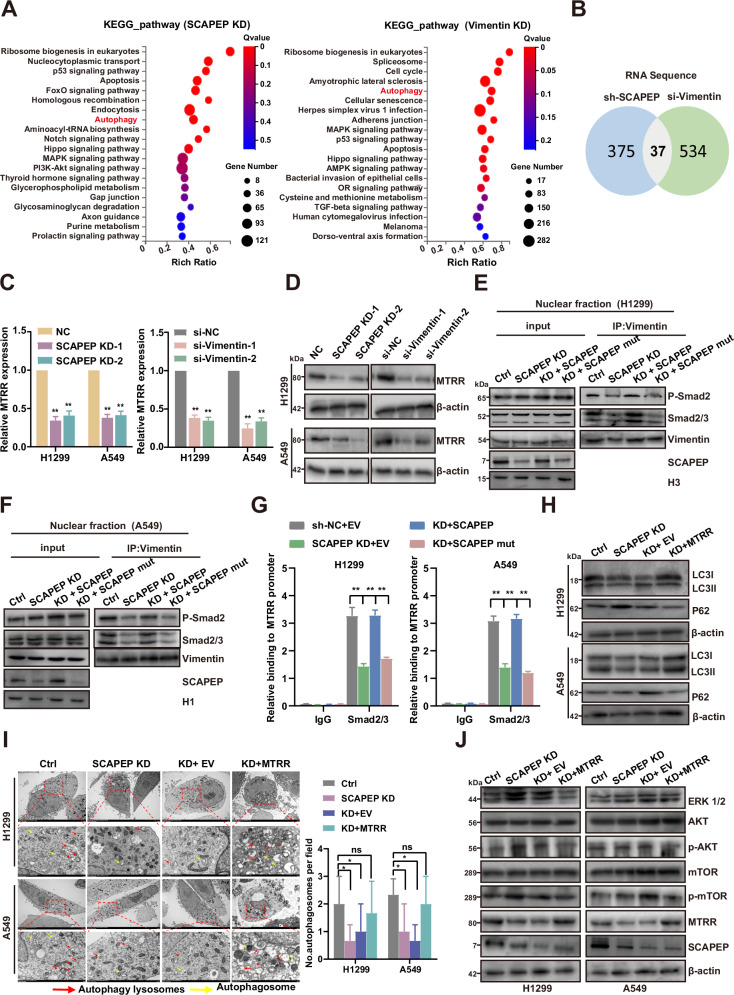
SCAPEP promotes autophagy occurrence in LUAD cells through co-regulating MTRR expression with vimentin. **A** Analysis of the KEGG pathway revealed the enrichment of differentially expressed genes in the signaling pathways of LUAD cells with knockdown of SCAPEP or vimentin. **B** The overlap of RNA-seq results from SCAPEP and vimentin knockdown experiments was visualized by a Venn diagram, thereby identifying the target genes of SCAPEP/vimentin. **C** qRT-PCR was conducted to assess the effect of SCAPEP or vimentin knockdown on the mRNA expression level of MTRR in LUAD cells. **D** Western blotting was used to measure the protein expression levels of MTRR in LUAD cells with knockdown of SCAPEP or vimentin. **E**, **F** Immunoprecipitation was performed on nuclear lysates derived from LUAD cells. For immunoprecipitation, anti-vimentin antibodies were employed, while immunoblotting was conducted using anti- vimentin, anti-p-Smad2, anti-Smad2/3, anti-SCAPEP and anti-histone H1 antibodies. **G** ChIP assays were conducted on chromatin fragments using antibodies specific to Smad2/3 or IgG, and the results were normalized to pre-immune normal IgG. The immunoprecipitated fractions were subsequently analyzed by qRT-PCR to evaluate their binding to the MTRR promoters. **H** Autophagy-associated proteins were detected by immunoblotting in four groups of LUAD cells: Ctrl, SCAPEP KD, KD + EV, and KD + MTRR. **I** Transmission EM showing autophagosomes in the four groups of LUAD cells mentioned above. Scale bars 1 μm. **J** In LUAD cells, western blotting was performed to detect AKT, p-AKT, m-TOR, p-mTOR, SCAPEP and MTRR, using β-actin as the internal control/reference. Differences between the groups were evaluated using Student’s *t* test or One-way ANOVA with Tukey’s Multiple Comparison test, *P < 0.05, **P < 0.01.

To further investigate the specific processes in which SCAPEP is involved in the malignant progression of LUAD, we conducted RNA-seq analysis using LUAD cell lines with stable SCAPEP knockdown, as well as performed KEGG pathway enrichment analysis on the differentially expressed genes following SCAPEP knockdown. The results demonstrated that these differentially expressed genes were predominantly enriched in signaling pathways associated with apoptosis, autophagy, proliferation, and metastasis (Fig. 7A). Simultaneously, we utilized LUAD cells with vimentin knockdown for RNA-seq analysis, and the KEGG pathway enrichment analysis revealed that the genes influenced by vimentin knockdown were predominantly enriched in signaling pathways related to cell cycle, autophagy, proliferation, metastasis, and apoptosis (Fig. 7A). Comparing the results of two KEGG pathway enrichment analyses, it is evident that the most significant shared impact of SCAPEP and vimentin on the malignant phenotype of LUAD is autophagy. Then, we performed a comprehensive analysis of the two RNA-Seq datasets obtained from separate knockdown experiments of SCAPEP and vimentin, and identified 37 target genes co-regulated by SCAPEP and vimentin (Fig. 7B–D). Among them, MTRR can induce autophagy by inhibiting the PI3K-AKT-mTOR signaling pathway, thus promoting ovarian cancer growth [37]. Therefore, we speculate that SCAPEP and vimentin may regulate the expression of MTRR to suppress the PI3K-AKT-mTOR signaling pathway and subsequently influence autophagy in LUAD cells. It is known that phosphorylated vimentin can interact with p-Smad2 and translocate to the nucleus, thereby regulating the transcription of downstream gene PD-L1 and consequently influencing the immune escape process of lung cancer cells [38]. Our previous results have demonstrated that SCAPEP can facilitate the phosphorylation of vimentin by CDK15. We further investigated and found that knockdown of SCAPEP attenuates the interaction between p-vimentin S56 and p-Smad2 within the Smad2/3 complex in the nucleus of LUAD cells, whereas overexpression of SCAPEP yields the opposite effect (Fig. 7E, F). Subsequently, to ascertain whether p-Smad2 triggers MTRR expression, we employed chromatin immunoprecipitation with anti-Smad2/3 antibodies and observed the detection of nuclear Smad2/3 in the promoter region of MTRR. Furthermore, the efficiency of nuclear Smad2/3 enrichment at MTRR was reduced in SCAPEP knockdown cells, whereas this phenomenon was restored in cells with SCAPEP re-expression (Fig. 7G). These results unveil that SCAPEP promotes vimentin phosphorylation, thereby facilitating the translocation of phosphorylated vimentin and p-Smad2 within the Smad2/3 complex into the nucleus to regulate MTRR expression.

Subsequently, we delved into whether SCAPEP regulates autophagy in LUAD cells by modulating MTRR expression. In the current study, we examined the expression of autophagy-related proteins in different groups and observed the number of autophagosomes in cells using transmission electron microscopy (Supplementary Fig. S4A, B). The results showed that, compared to the control group, LUAD cells with SCAPEP knockdown exhibited downregulation of LC3-II expression and upregulation of P62 expression, accompanied by a significant decrease in the number of autophagosomes. However, in cells re-expressing SCAPEP, the expression of LC3-II was restored, P62 expression was downregulated, and the number of autophagosomes was significantly recovered. Notably, these phenomena were not restored in cells re-expressing SCAPEP mut. These findings indicate that SCAPEP promotes autophagy in LUAD cells. Furthermore, we validated that vimentin and MTRR also promote autophagy in LUAD cells (Supplementary Fig. S4C–F). Next, we conducted rescue experiments using four groups of LUAD cells: Ctrl, SCAPEP KD, KD + EV, and KD + MTRR. The results demonstrated that, compared to the control group, LUAD cells with SCAPEP knockdown exhibited significant alterations in autophagy-related proteins and autophagosomes, and these aberrations were restored upon re-expression of MTRR (Fig. 7H, I). It is known that the PI3K-AKT1 signaling pathway is regulated by mTOR (PI3K-AKT-mTOR), which can inhibit autophagy [39, 40]. Additionally, MTRR can influence autophagy in LUAD cells by modulating the PI3K-AKT-mTOR signaling pathway. Similarly, our results suggested that SCAPEP also inhibited the PI3K-AKT-mTOR signaling pathway in LUAD cells (Supplementary Fig. 4G). Therefore, we continued to explore whether SCAPEP regulates the PI3K-AKT-mTOR signaling pathway through MTRR. To evaluate the expression of MAPK, ERK1/2, AKT, and mTOR, western blot analysis was performed on the four groups of cells mentioned above. Comparative analysis revealed that SCAPEP knockdown resulted in a substantial increase in the expression levels of ERK1/2, p-AKT, and p-mTOR, in contrast to the control group. However, upon re-expression of MTRR, the expression of these proteins was suppressed (Fig. 7J). These observations strongly suggest that SCAPEP regulates autophagy in LUAD cells via the involvement of MTRR.

## Discussion

In recent years, advances in genome-wide translation profiling and ribosome footprinting have revealed numerous non-canonical ORFs in transcripts previously annotated as noncoding RNAs, and an increasing number of circRNA-encoded micropeptides have been shown to exert important functions in cancer [41, 42]. For instance, C-IGF1R, encoded by cIGF1R, acts as a molecular switch between mitophagy and apoptosis in lung cancer by disrupting Parkin-mediated polyubiquitylation of VDAC1 [43], whereas GSPT1-238aa, derived from circGSPT1, modulates autophagy via the PI3K/AKT/mTOR pathway and functions as a tumor suppressor in gastric carcinoma [44]. In contrast to these micropeptides, SCAPEP identified in our study recruits the kinase CDK15 to phosphorylate vimentin at Ser56, thereby upregulating MTRR and activating autophagy. To our knowledge, SCAPEP is the first circRNA-encoded peptide reported to regulate autophagy through a “peptide–kinase–cytoskeletal protein” ternary complex. Functionally, SCAPEP is highly expressed in LUAD tissues, associates with larger tumor size, lymph node metastasis, advanced stage and poor prognosis and promotes LUAD cell proliferation and metastasis in vitro and in vivo, at least in part through this autophagy-regulatory mechanism (Fig. 8).

The involvement of cytoskeletal proteins in autophagy has long been recognized, but the underlying mechanisms remain incompletely understood. Vimentin, a type III intermediate filament protein widely expressed in mesenchymal cells, regulates cell shape, migration and wound healing through EMT-related signaling and fibroblast proliferation [45, 46], and its upregulation is tightly linked to tumor invasion and metastasis [47]. Vimentin has also been implicated in the spatial organization of autophagosomes and lysosomes via mTORC1 signaling [48], and can form an autophagy-inhibitory complex with 14-3-3 and Beclin 1 in an AKT-dependent manner to suppress autophagy [44, 49]. In our study, knockdown of either SCAPEP or vimentin reduced LC3-II levels, increased accumulation of the autophagic substrate p62 and markedly decreased autophagosome number, supporting a positive role for the SCAPEP–vimentin axis in autophagy induction in LUAD cells. Notably, SCAPEP binds vimentin without altering its total RNA or protein abundance, but significantly enhances vimentin phosphorylation, indicating a post-translational mechanism by which SCAPEP rewires vimentin function toward promoting autophagy.

CDK15, a member of the PFTAIRE subfamily of CDKs (CDK14/CDK15), has been reported to promote tumorigenesis by phosphorylating PAK4 [50]. Given that total vimentin levels remained unchanged upon SCAPEP overexpression or knockdown, we focused on vimentin phosphorylation. Distinct phosphorylation events on vimentin serine residues are known to control diverse biological processes; for example, PLK1-mediated phosphorylation at Ser339 facilitates nuclear localization of p-Smad2 and upregulation of PD-L1, thereby promoting immune escape in metastatic LUAD [38]. Here, co-immunoprecipitation experiments and in vitro kinase assays demonstrated that CDK15 directly phosphorylates vimentin, and phosphoproteomic analysis identified Ser56 as a major CDK15 target site. Ser56 is the only serine residue immediately followed by a proline in vimentin, consistent with the proline-directed substrate specificity of CDKs [36], and mutation of Ser56 to alanine markedly impaired vimentin phosphorylation by CDK15. Importantly, SCAPEP strengthened the interaction between CDK15 and vimentin and promoted Ser56 phosphorylation in a dose-dependent manner, suggesting that SCAPEP acts as a scaffold-like micropeptide that facilitates assembly of the SCAPEP/CDK15/vimentin complex. Our data further indicate that Ser56 phosphorylation of vimentin leads to increased MTRR expression. MTRR is required for maintaining methionine synthase activity and has been implicated in DNA synthesis and carcinogenesis; its silencing inhibits growth and cisplatin resistance in ovarian cancer by reducing autophagy and inducing apoptosis [37]^.^ Consistent with this, MTRR overexpression in SCAPEP-knockdown LUAD cells rescued autophagy defects and attenuated the suppression of malignant phenotypes, supporting a model in which SCAPEP promotes LUAD progression by enhancing CDK15-mediated vimentin Ser56 phosphorylation and MTRR upregulation to drive pro-tumorigenic autophagy.

A key mechanistic link between the SCAPEP/CDK15/vimentin complex and MTRR transcription involves the nuclear translocation of phosphorylated vimentin and Smad2/3. It has been reported that phosphorylated vimentin can interact with p-Smad2 and facilitate its nuclear localization, where the complex binds the PD-L1 promoter and contributes to immune evasion in LUAD38. Building on this concept, we show that SCAPEP promotes the interaction between phospho-vimentin (Ser56) and p-Smad2 within the Smad2/3 complex in the nucleus, thereby enhancing Smad2/3 recruitment to the MTRR promoter. ChIP assays confirmed Smad2/3 enrichment at the MTRR promoter, which was diminished in SCAPEP-knockdown cells and restored upon SCAPEP re-expression. Together with the MTRR rescue experiments, these data support a model in which SCAPEP, via CDK15-dependent vimentin phosphorylation, couples cytoskeletal signaling to TGF-β/Smad transcriptional output to drive MTRR expression and autophagy. Furthermore, we show that SCAPEP inhibits the PI3K–AKT–mTOR pathway in an MTRR-dependent manner, consistent with the well-established role of PI3K–AKT–mTOR signaling as a negative regulator of autophagy [39, 40]. Thus, SCAPEP exerts coordinated control over both cytoskeletal and metabolic/autophagy pathways to sustain LUAD cell survival and aggressiveness.

Our observation that SCAPEP-driven autophagy promotes LUAD progression appears, at first sight, to conflict with reports in which autophagy restrains tumorigenesis, including KRAS-driven LUAD models discussed in the Introduction. This apparent discrepancy can be reconciled by the now well-recognized dual and context-dependent roles of autophagy in cancer. Large-scale genetic and animal studies, together with recent comprehensive reviews, have established that autophagy may function either as a tumor suppressor or as a tumor promoter, depending on tumor stage, oncogenic background and microenvironmental context [14, 51]. At early stages of tumor development, autophagy exerts tumor-suppressive effects by clearing damaged organelles and misfolded proteins, thereby limiting oxidative stress and preserving genomic integrity [14]. In contrast, once tumors are established, many cancers become highly dependent on autophagy to satisfy elevated metabolic demands, adapt to hypoxia and nutrient deprivation, and withstand therapeutic stress—a phenomenon of “autophagy addiction” that has been particularly well documented in Ras/Braf-driven tumors [51, 52]. In KRAS-driven lung cancer models, genetic ablation of core autophagy genes such as Atg5 or Atg7 accelerates the formation of early hyperplastic lesions but markedly impairs progression to malignant adenocarcinoma or diverts tumor fate toward benign oncocytomas, highlighting a stage-dependent switch from tumor-suppressive to tumor-supportive autophagy [53, 54]. Oncogenic Ras/KRAS has also been shown to upregulate basal autophagy to maintain mitochondrial function and oxidative metabolism, and loss of autophagy in this context forces tumor cells into a less aggressive, metabolically compromised state [53, 55]. Our data are consistent with this framework: SCAPEP is predominantly expressed in established LUAD and activates an autophagy program that sustains tumor cell proliferation, survival and metastatic potential. By engaging the SCAPEP/CDK15/vimentin/MTRR axis and suppressing PI3K–AKT–mTOR signaling, SCAPEP appears to skew autophagy toward a pro-tumor outcome tailored to the metabolic and stress-adaptive requirements of advanced LUAD cells. Thus, rather than contradicting previous reports, our findings reinforce the concept that the biological consequences of autophagy are highly contingent on tumor stage, genetic context and the specific upstream pathways that elicit autophagic flux ^53, 54^.

In summary, we identify SCAPEP as a novel circRNA-encoded micropeptide translated from circRNA_0065214 that is significantly upregulated in LUAD tissues and promotes LUAD cell proliferation, metastasis and autophagy in vitro and in vivo. Mechanistically, SCAPEP forms a ternary complex with CDK15 and vimentin, enhances CDK15-mediated vimentin Ser56 phosphorylation and MTRR upregulation, and thereby activates pro-tumorigenic autophagy. These findings uncover a previously unrecognized “peptide–kinase–cytoskeletal protein” axis linking circRNA-derived oncogenic peptides to autophagy control, and suggest that targeting the SCAPEP/CDK15/vimentin/MTRR pathway may represent a promising therapeutic strategy in lung adenocarcinoma.

## Materials and methods

### Tissue specimens and cell lines

Thirty pairs of fresh LUAD tissues and adjacent non-tumor lung tissues were procured from Huzhou Central Hospital, located in Zhejiang Province, China. The tissue chip consisting of 90 LUAD tissue samples was obtained from AiFang Biological (HN20250401). The tissue chip provides clinicopathological and survival information relevant to the present study. Our study was approved by the Medical Ethics Committee of Huzhou Central Hospital and was conducted in compliance with the ethical guidelines outlined in the Declaration of Helsinki of the World Medical Association. Written informed consent was obtained from all patients prior to their participation in the study. The normal human lung epithelial cell line 16HBE, various LUAD cell lines (H1299, A549, PC9, HCC827, H1975), and human embryonic kidney HEK-293T cells were obtained from the American Type Culture Collection (ATCC). These cells were cultured in complete accordance with ATCC guidelines, using Dulbecco’s modified Eagle medium (DMEM) supplemented with 10% FBS (Gibco, Waltham, MA, USA) and maintained at 37 °C with 5% CO_2_. Monthly testing of Mycoplasma infection was conducted on these cells by PCR.

### RNA extraction and reverse transcription-qPCR

Total RNAs were extracted from the tissues and cultured cells using TRIzol reagent (Invitrogen, Carlsbad, CA, USA) following the manufacturer’s protocol. Reverse transcription of approximately 1 μg of total RNA to cDNA was performed using a cDNA synthesis kit, and qPCR was carried out using a SYBR Green PCR kit (Takara, Kusatsu, Japan). All reactions were performed in triplicate on a CFX96TM Touch Real-Time PCR System (Bio-Rad, Hercules, California, USA). Comparative quantification was performed using the 2^−ΔCt^ method. The reactions were performed in triplicate in three independent experiments, and the results were normalized to those of GAPDH. The primers used in this study are listed in Supplementary Table S2.

### Subcellular fractionation

Subcellular fractionation was performed to obtain the cytoplasmic and nuclear fractions using NE-PER Nuclear and Cytoplasmic Extraction Reagents (Thermo Fisher Scientific, Waltham, MA, USA) in accordance with the manufacturer’s instructions. GAPDH was used as the cytoplasmic endogenous control, while the small nuclear RNA U6 was used as the nuclear endogenous control.

### LC-MS/MS identification of SCAPEP

The identification of SCAPEP peptides by LC-MS/MS was conducted as follows: Following extraction with RIPA buffer from NSCLC tissues and cells, proteins were separated by SDS-PAGE and visualized via Coomassie Brilliant Blue staining. Subsequently, lower molecular weight protein bands (<15 kDa) were excised, divided into three fractions, and subjected to in-gel digestion. The resulting peptides were analyzed using a QExactive mass spectrometer coupled to a nano-LC system (AdvanceLC, Michrom Inc.) via a nano-electrospray source, with the column oven maintained at 37 °C (AMR Inc.). After being dissolved in 0.1% TFA and 2% acetonitrile, samples were loaded onto a pre-column (L-column micro; 0.3 mm inner diameter, 5 mm length; CERI, Japan) and separated on an in-house-packed 20-cm column (100 μm inner diameter; CERI, Japan). Separation was achieved using a linear gradient of 5–35% B over 90 min, followed by an increase to 95% B in 1 min and a 10-min hold at 95% B (Mobile phase A: 0.1% formic acid, 2% acetonitrile; B: 0.1% formic acid, 90% acetonitrile), at a constant flow rate of 250 nL/min. The mass spectrometer was operated in data-dependent acquisition mode, with full MS scans acquired at a resolution of 70,000 at m/z 200. The acquired MS/MS spectra were searched using the SEQUEST HT algorithm against a custom database generated from a three-frame (forward only) translation of ncRNA sequences (transcripts >5 amino acids). The search parameters included: trypsin as the enzyme, up to 2 missed cleavages, carbamidomethylation of cysteine as a fixed modification, and oxidation of methionine and N-terminal acetylation as variable modifications. A false discovery rate (FDR) of less than 1% (*q*-value < 0.01) was estimated using the Percolator algorithm integrated into the Proteome Discoverer suite.

### Western blotting analysis

Proteins were separated by SDS-PAGE, followed by transfer to a nitrocellulose membrane (Bio-Rad). The membranes were blocked with 5% nonfat milk and incubated with primary antibodies. The resulting immune complexes were detected using an enhanced chemiluminescence reagent (Pierce, Waltham, MA, USA). Antibodies against SCAPEP were specifically tailored using ABClonal software. Information on the antibodies used is provided in Supplementary Table S4.

### RNA stability analysis

LUAD cells were exposed to 5 μg/mL actinomycin D and harvested at specified time intervals. Total RNA was extracted using TRIzol reagent (Invitrogen), followed by quantitative reverse transcription-PCR to determine circ_0065214 and SCAP mRNA levels. The reference gene GAPDH was used for normalization.

### Dual-luciferase reporter assay

The wild-type or truncated sequences of the IRES of circ_0065214 were inserted into a vector containing a luciferase reporter gene and transfected separately into HEK293T cell plasmids. After 48 h of transfection, luciferase reporter gene detection was performed using a dual-luciferase reporter gene assay system, according to the manufacturer’s instructions (Promega, Madison, WI, USA). Relative luciferase activity was normalized to Renilla luciferase activity.

### Immunofluorescence staining

LUAD cells were seeded onto 24-well plates containing cell-coated glass slides. After stable adhesion and growth, cells were transfected with plasmids (ORF1-Flag, ORF1 mut-Flag, and vector) using the PolyJet reagent. After 24 h, the cells were fixed with 4% paraformaldehyde, permeabilized with 0.5% Triton X-100 for 10 min, and blocked with 5% BSA for 1 h. Subsequently, immunostaining was performed using primary antibodies specific to the FLAG protein (diluted 1:100) and appropriate secondary antibodies (goat anti-rabbit IgG [H + L] 488). To analyze protein localization, immunostaining was performed with primary antibodies targeting SCAPEP (diluted 1:200), vimentin (diluted 1:200), or CDK15 (diluted 1:200), followed by appropriate secondary antibodies. Nuclear staining was accomplished using DAPI (4’,6-diamidino-2-phenylindole, Thermo Fisher Scientific). The images were acquired using a confocal microscope (FV1200; Olympus, Tokyo, Japan).

### Plasmids, siRNAs, lentiviral construction, and stable transfection

The human MTRR-overexpression vector and the corresponding control plasmid were procured from GeneLily Biotechnology (Shanghai, China). SCAPEP expression vectors equipped with Flag markers (ORF1-Flag and ORF1 Mut-Flag) and full-length and site-directed mutants (S56R) of vimentin were provided by OBiO Technology (Shanghai, China). Human-specific small interfering RNAs (siRNAs) used for brief silencing of vimentin and CDK15 expression were produced by GenePharma Corporation (Shanghai, China). Lipofectamine 2000 (Invitrogen) was used for transfection, according to the manufacturer’s instructions. The transfection efficacy of the cells in each group was evaluated after 48 h using real-time PCR. Lentivirus vectors bearing shRNAs targeting SCAPEP were generated by GenePharma (Shanghai, China), and stable cell lines were obtained by selection with puromycin. The small interference sequence information used in this paper is shown in supplementary Table S3.

### Cell proliferation and colony-formation assays

Cell proliferation assays were performed using Cell Counting Kit-8 (CCK-8; Vazyme, Nanjing, China). The cells were seeded at a density of 1000 cells per well in 96-well plates, and 10 µL of CCK-8 solution was added to each well. Subsequently, the plates were incubated at 37 °C for 2 h and the absorbance was determined at 450 nm. The experimental procedure was repeated at least three times, and each measurement was performed in triplicate to ensure accuracy. For colony-formation experiments, the cells were trypsinized and plated in 6-well plates at a density of 1000 cells per well. After 14 days, the cells were stained with a solution containing 0.5% crystal violet. The colonies were visually assessed using an inverted microscope (Olympus).

### Cell migration and invasion assays

For the migration experiment, 1 × 10^4^ cells were seeded into the upper chamber of a transwell plate (BD Biosciences, Franklin Lakes, NJ, USA) containing serum-free DMEM. For the invasion assay, 1 × 10^5^ cells were introduced into the upper compartment of each Matrigel-coated insert. The lower chamber was filled with DMEM supplemented with 10% FBS. Following incubation at 37 °C, the non-migrating cells in the upper chamber were removed with a cotton swab and fixed using methanol, and the cells adhering to the lower membrane were stained with a 0.5% crystal violet solution. Visualization and enumeration of cells migrating to or invading the membrane were conducted using an inverted microscope (Olympus).

### Immunoprecipitation assay

After trypsinizing the target cells, the cell pellet was obtained by low-speed centrifugation and washed once with PBS. Subsequently, the cells were treated with an appropriate lysis buffer containing a protease inhibitor (Life Technologies, Carlsbad, CA, USA; 250 mM NaCl, 50 mM Tris pH 8, 5 mM EDTA, 0.8% NP40, 1 mM DTT, 5% glycerol, and 50 mM NaF) and placed on ice for 30 min for lysis. The supernatant was collected by centrifugation at 12,000 × *g* for 15 min. The supernatant was gently rotated with a specific antibody against the target protein at 4 °C overnight. Subsequently, the antibody-protein complex was incubated with 30 μL of protein A/G beads (Thermo Fisher Scientific) at room temperature for 2 h. Protein complexes were isolated by SDS-PAGE for western blotting analysis.

### In vitro kinase assay

For in vitro kinase assays, pGEX-vimentin WT(S56A)-GST plasmids were constructed and transformed into *E. coli* BL21 for cultivation. Protein expression was induced with IPTG when the cultures reached the logarithmic growth phase. Recombinant CDK15 protein for in vitro assays was purchased from ICE BioScience (Catalog No.: S2401F-H21GH2; Beijing, China). Active recombinant CDK15 (500 ng) was incubated with either wild-type (WT) or mutant PAK4 recombinant protein (400 ng) in the presence of 250 μM ATP. The kinase reaction was carried out in kinase buffer (20 mmol/L HEPES, pH 7.4, 10 mmol/L MgCl_2_, 5 mmol/L EGTA, 150 mmol/L NaCl, 20 mmol/L β-glycerol phosphate) for 30 min at 30 °C and was terminated by adding 6× SDS sample buffer. Proteins were separated by SDS-PAGE and analyzed by Western blot.

### Chromatin immunoprecipitation (ChIP)

An EZ ChIP kit (Merck Millipore, Darmstadt, Germany) was used to conduct ChIP assays, following the manufacturer’s instructions. Initially, 5 × 10^6^ cells were fixed at room temperature with 4% paraformaldehyde (Sigma) for 10 min for cross-linking, followed by the addition of a 1/10 volume of 1.25 M glycine and incubation at room temperature for 5 min to halt fixation. Ultrasound treatment was then performed for 5 min (on for 30 s and off for 30 s). Each immunoprecipitation utilized 200 μg of protein-chromatin complex. The antibody-protein complex was isolated using pre-blocked Dynabead Protein G (Invitrogen). Precipitated DNA was analyzed by qRT-PCR using primers targeting the MTRR promoter region. The enrichment value was calculated, normalized to the input, and expressed as a ratio relative to IgG.

### Transmission electron microscopy

LUAD cells were collected after digestion with 0.5% trypsin, washed once with PBS, and fixed in a fixation solution. Subsequently, the cells were treated with 2.5% glutaraldehyde in PBS and fixed with 1% osmium tetroxide. After dehydration using a series of ethanol gradients (30–90%) and embedding in epoxy resin, ultrathin sections (80 nm) were stained with 2% uranyl acetate and lead citrate and then observed under an H7650 TEM electron microscope (Hitachi, Tokyo, Japan).

### Immunohistochemistry

Subcutaneous xenograft samples were processed by embedding in paraffin and subsequent sectioning. Subsequently, the samples underwent overnight incubation at 4 °C with either anti-Ki67 or anti-SCAPEP antibodies. The tissue sections were then subjected to a 30-min incubation at 37 °C with biotinylated goat anti-rabbit immunoglobulin (1:100). Next, the samples were incubated with 3,3’-diaminobenzidine. Two independent pathologists assessed all the immunohistochemical samples. Finally, images were captured using an inverted confocal laser-scanning microscope.

### Animal experiments

In vivo animal experiments were approved by the Ethics Committee of Huzhou Central Hospital (202311006). Four-week-old BALB/c-nu mice were purchased from GemPharmatech Co., Ltd. (Jiangsu, China). Mice were housed in SPF-grade cages (≤5 mice/cage) under standard specific pathogen-free (SPF) conditions with air filtration (22 ± 2 °C, 12 h light/12 h dark cycle) and provided food and water ad libitum. To conduct in vivo tumorigenic experiments, 1.5 × 10^6^ LUAD cells from each experimental group (control, SCAPEP KD, KD + SCAPEP, and KD + SCAPEP mut) were subcutaneously injected into the lateral ventral area of male nude mice at 4 weeks of age (each group *n* = 5). Each group of mice was randomly assigned. Weekly monitoring of the tumor size in mice was performed post-transplantation. After 8 weeks, the mice were euthanized, and the tumors were fixed for histological analysis. Furthermore, 2 × 10^6^ cells from the experimental groups were injected into the tail vein of nude mice for in vivo transfer testing (each group *n* = 3). Approximately 2 months later, the mice were euthanized, and lung tissues were fixed, embedded in paraffin, and sectioned for histopathological examination. All experimental procedures complied with the protocols approved by the Experimental Animal Protection Committee of Huzhou Central Hospital.

### Statistics

Unless otherwise stated in the figure legend, all experiments were conducted at least three times. All experimental data were generated using blinded analysis. A two-tailed paired Student’s *t* test was used to compare two groups. Survival curves were generated using the Kaplan-Meier method and compared using the log-rank test. Statistical analyses were performed using GraphPad Prism 8 software (GraphPad, San Diego, CA, USA). Data are expressed as the mean ± SD unless specified otherwise. Statistical significance was set at *P* < 0.05.

## Supplementary information

Supplementary Figure and Figure Legend

Full Western Blots

Supplementary Tables 1-5

Supplementary Table 6

## Data Availability

The circRNA chip microarray sequencing data used in this paper were downloaded from Gene Expression Omnibus (GEO) with accession numbers GSE101586. (https://www.ncbi.nlm.nih.gov/gds/?term=GSE101586).
