# Supplementary material for: Micropeptide SCAPEP triggers lung adenocarcinoma tumorigenesis via regulating autophagy by promoting CDK15-mediated phosphorylation of vimentin
Source: Cell Death Dis. 2026 May 6;17(1):602. doi: 10.1038/s41419-026-08767-1 (PMC13315923; doi:10.1038/s41419-026-08767-1)
Supplement: Supplementary file 1 — Supplementary Figure and Figure Legend [file 41419_2026_8767_MOESM1_ESM.docx]

**Supplementary Figure and Figure Legend**


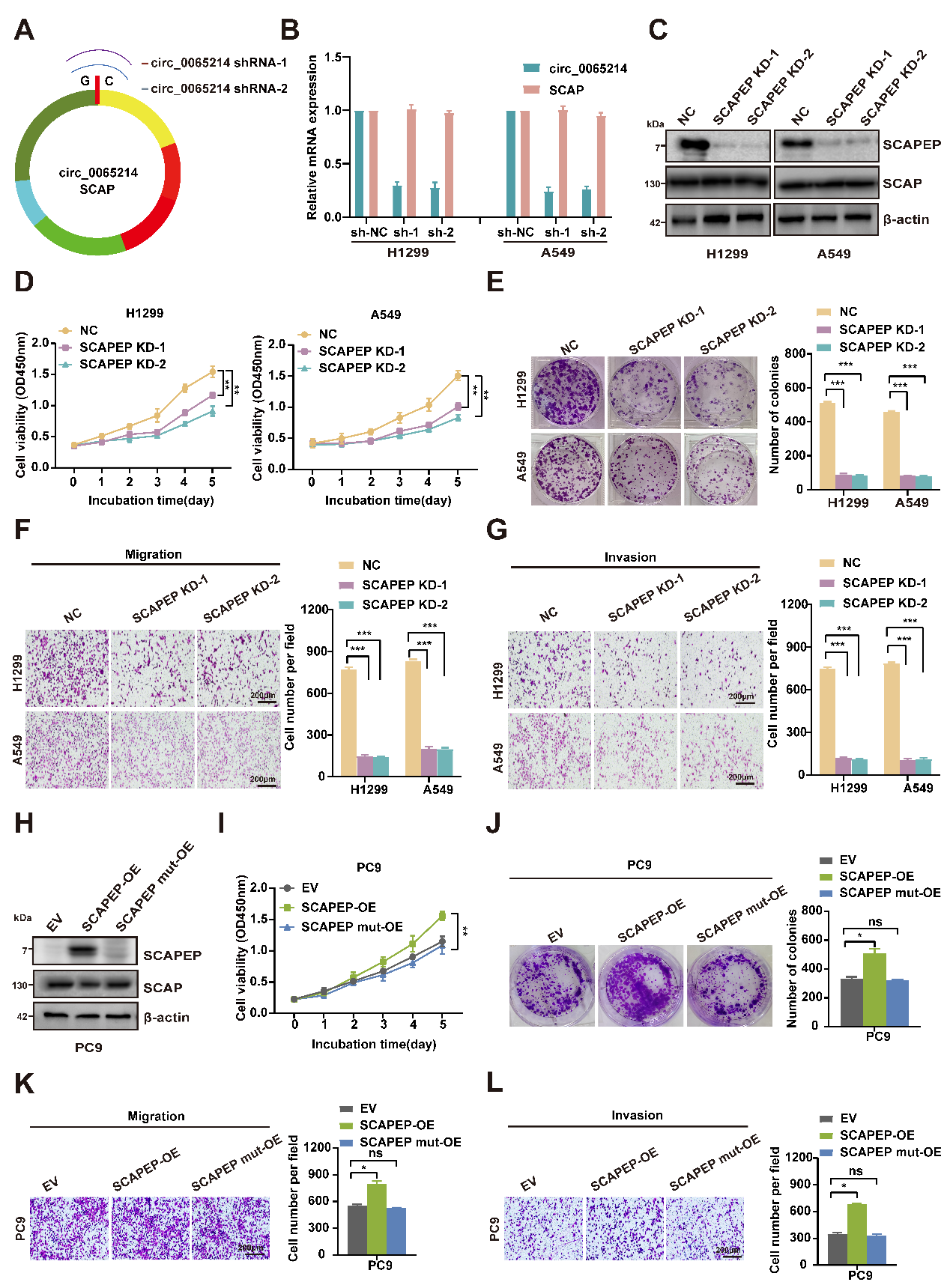


**Figure S1.** SCAPEP promotes proliferation and metastasis of LUAD cells.

(A) Schematic representation of the designed shRNA targeting the circularization site of circ_0065214. (B) The RNA expression levels of circ_0065214 and SCAP in LUAD cells with knocked down circ_0065214 were detected using qRT-PCR. (C) The protein expression levels of SCAPEP and SCAP in LUAD cells with knocked down SCAPEP were assessed using western blot analysis. (D, E) CCK-8 and colony formation assay to detect the proliferative activity of LUAD cells with SCAPEP stable knockdown. (F, G) Transwell assay was performed to evaluate the migration ability of LUAD cells with stable knockdown of SCAPEP. scale bars, 200 nm. H) western blot analysis was conducted to assess the transfection efficiency of SCAPEP and SCAPEP mut in LUAD cells. (I, J) The proliferative activity of LUAD cells transfected with SCAPEP or SCAPEP mut was measured by CCK-8 and colony-formation assay. (K, L) Transwell assay was performed to assess the migration ability of LUAD cells transfected with either SCAPEP or SCAPEP mut, scale bars, 200 nm. Data are represented as mean ± SD. Differences between the groups were evaluated using Student’s t-test, ns indicates no significance, *P <0 .05, **P <0 .01, ***P < 0.001.


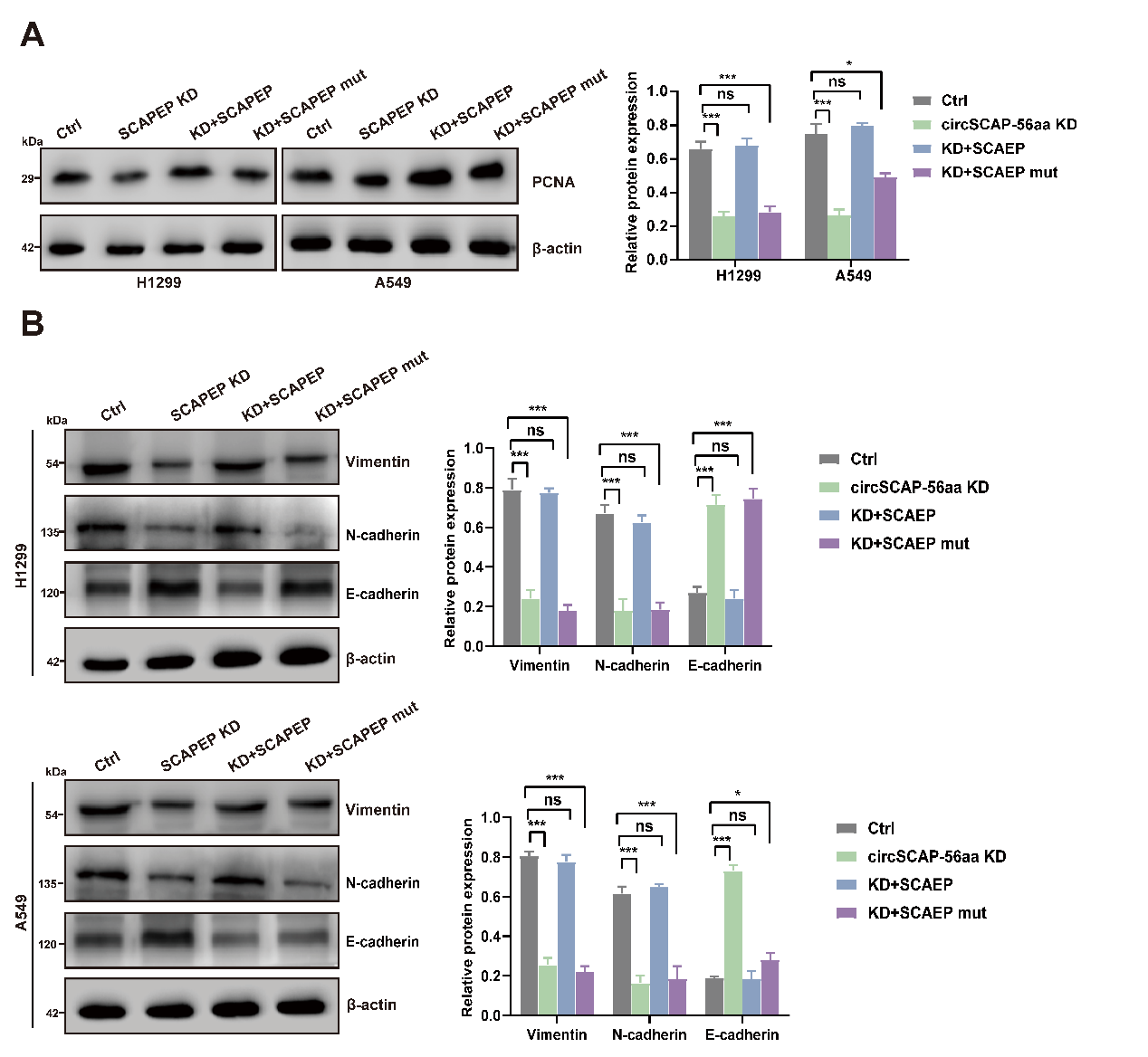


**Figure S2**. The expression of cell proliferation and metastasis markers in cells with different expression levels of SCPEP was detected by western blot.

(A)Western blot analysis was used to examine the expression of the cell proliferation marker PCAN. (B) Western blot analysis was used to examine the expression of the cell metastasis marker Vimentin, E-cadherin and N-cadherin. *P <0 .05; ***P < 0.001; ns, not significant. Data are represented as mean ± SD. Differences between the groups were evaluated using Student’s t-test, ns indicates no significance, *P <0 .05, ***P < 0.001.

**
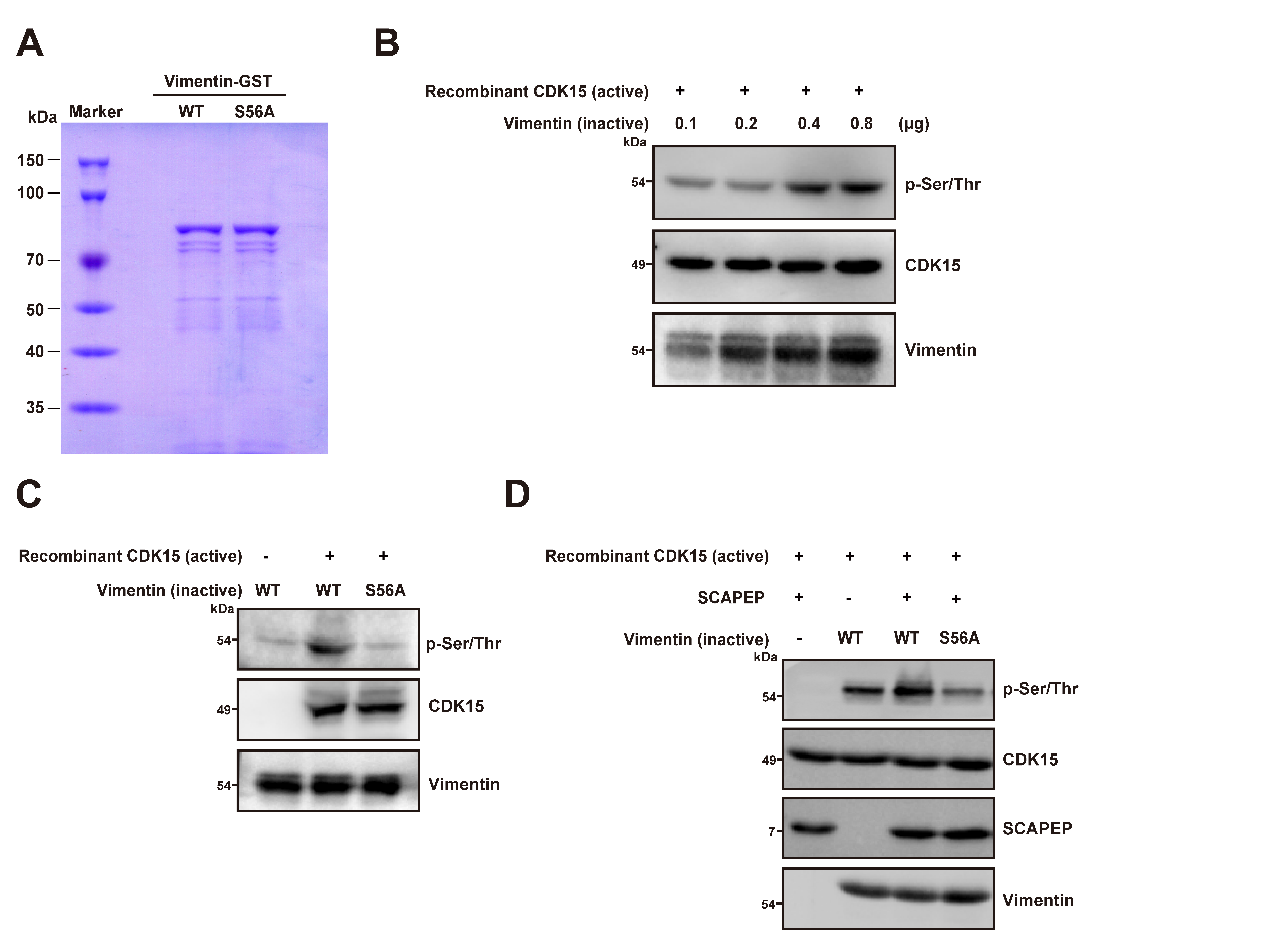
**

**Figure S3**. **CDK15 phosphorylates vimentin at Ser56 in vitro.**

(A) Purified recombinant vimentin proteins used for in vitro kinase assays. Wild-type (WT) and S56A mutant vimentin fused to GST were expressed and purified. (B) In vitro kinase assay showing phosphorylation of vimentin by CDK15 in a dose-dependent manner. Increasing amounts of active CDK15 (0.1–0.8 µg) were incubated with inactive vimentin. (C) Active CDK15 was incubated with either WT or S56A vimentin. Phosphorylation signals were detected by western blot. (D) In vitro kinase assays were performed with active CDK15, SCAPEP, and either WT or S56A vimentin. Phosphorylation levels were assessed with an anti-p-Ser/Thr antibody.

**
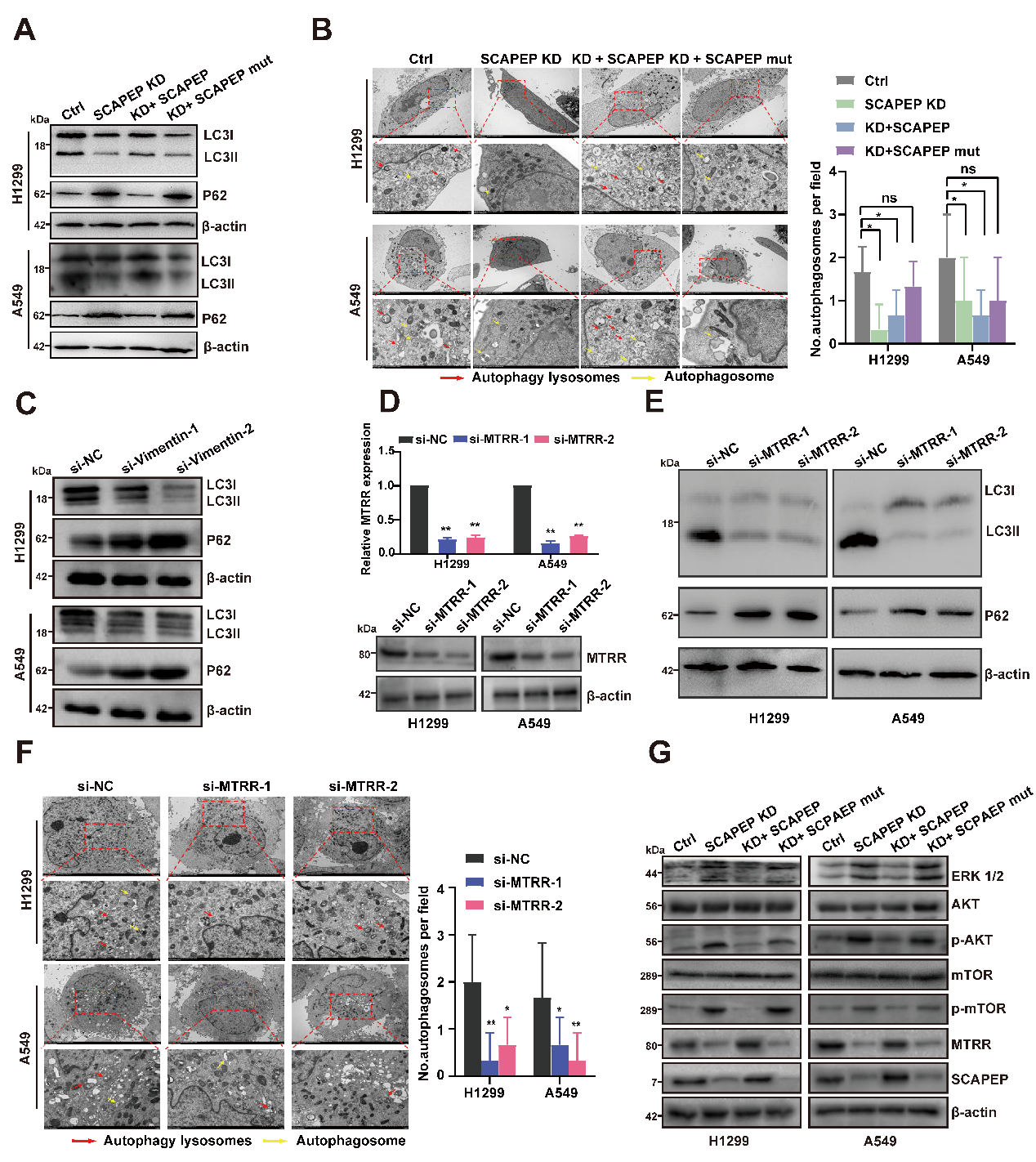
**

**Figure S4.** SCAPEP promotes autophagy in LUAD cells by regulating the PI3K-Akt-mTOR signaling pathway**.**

（A) The protein expression levels of autophagy-related marker proteins LC3I, LC3II, and P62 were detected by western blot in four groups of LUAD cells (Ctrl, SCAPEP KD, KD+SCAPEP, and KD+SCAPEP mut). (B) Transmission EM showing the presence of autophagosomes in four groups of LUAD cells (Ctrl, SCAPEP KD, KD+SCAPEP, and KD+SCAPEP mut). Scale bars 1 μm.（C）Western blot analysis of the expression levels of autophagy-related marker proteins after vimentin knockdown in lung cancer cells. (D）The knockdown efficiency of MTRR in LUAD cells was assessed by qRT-PCR and western blot after interfering MTTR with siRNAs. (E)The expression of autophagy-associated marker proteins was detected in MTRR knockdown cell lines using western blotting. (F)Transmission EM showing the presence of autophagosomes in MTRR knockdown cell lines. Scale bars 1 μm. (G) Western blot analysis provided evidence that SCAPEP modulates the PI3K-Akt-mTOR signaling pathway in LUAD cells. Data are represented as mean ± SD. Differences between the groups were evaluated using Student’s t-test, ns indicates no significance, *P <0 .05, **P <0 .01.
