# Supplementary material for: Micropeptide SCAPEP triggers lung adenocarcinoma tumorigenesis via regulating autophagy by promoting CDK15-mediated phosphorylation of vimentin
Source: Cell Death Dis. 2026 May 6;17(1):602. doi: 10.1038/s41419-026-08767-1 (PMC13315923; doi:10.1038/s41419-026-08767-1)

Figure 2D

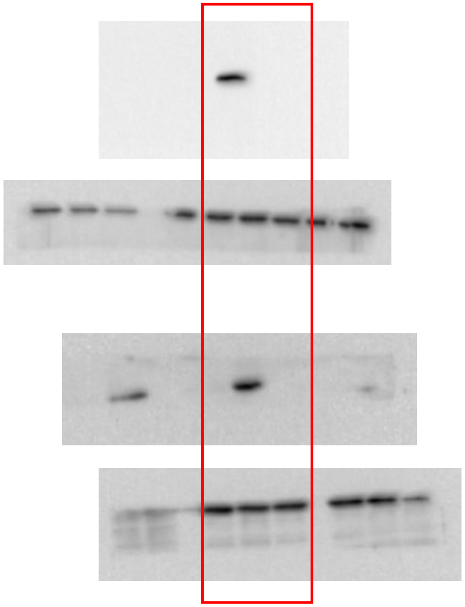

Figure 3C

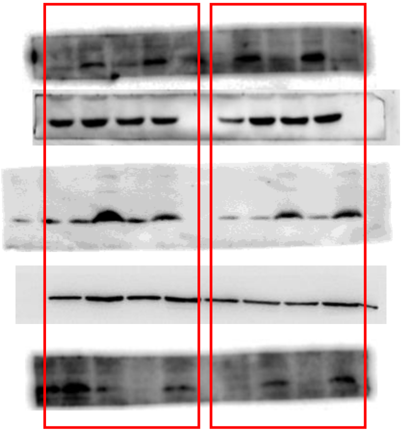

Figure 3E

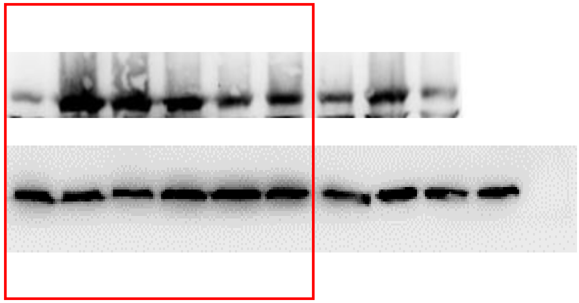

Figure 5F

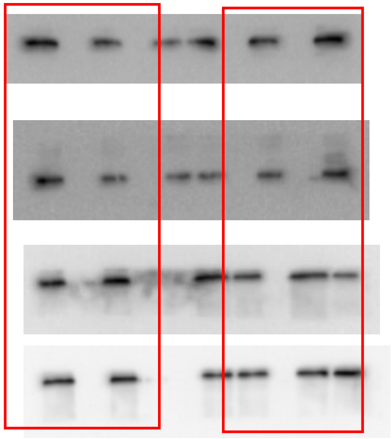

Figure 5J

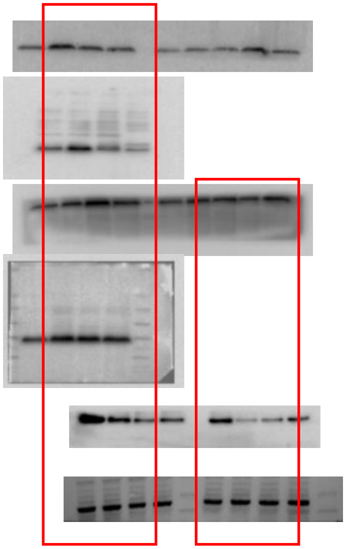

Figure 5I

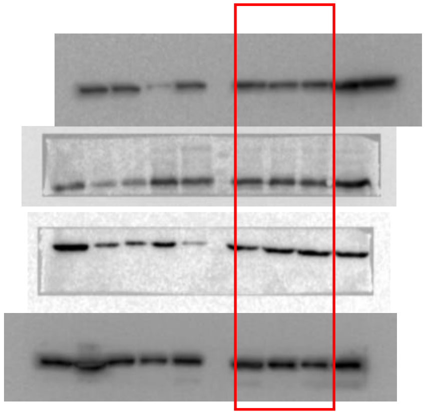

Figure 5L

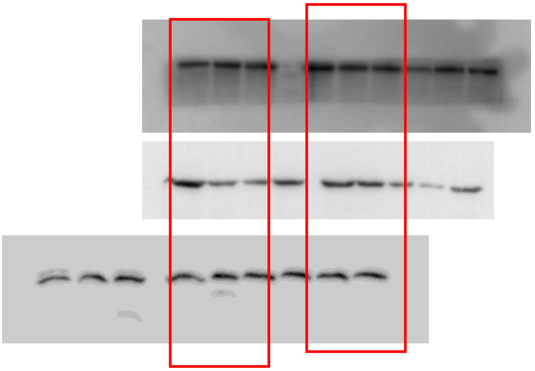

Figure 6B

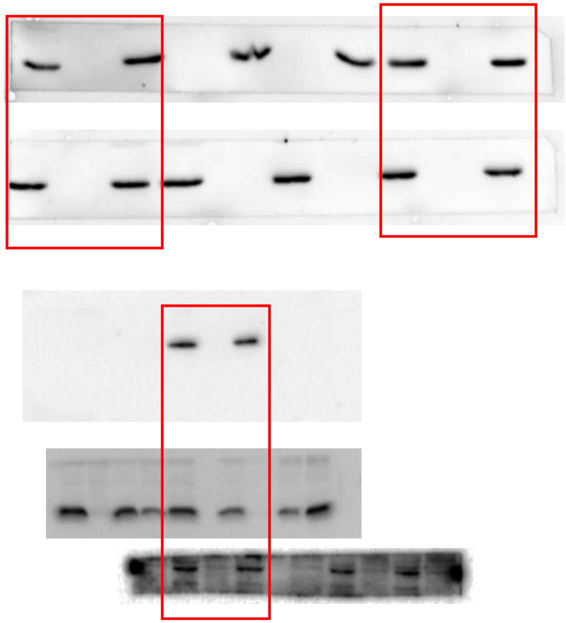

Figure 6E

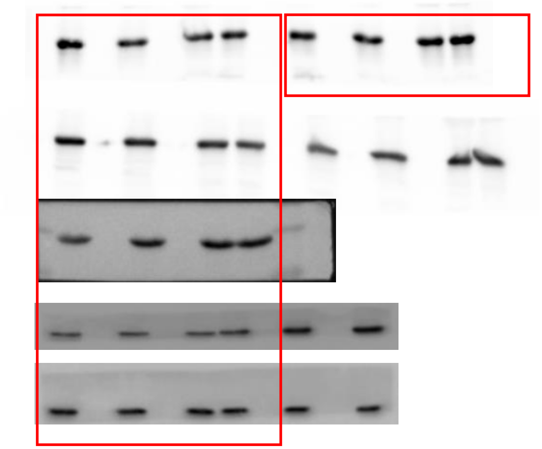

Figure 6F

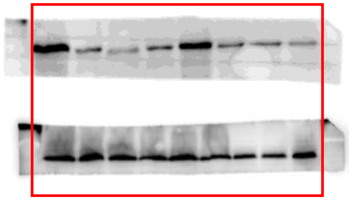

Figure 6G

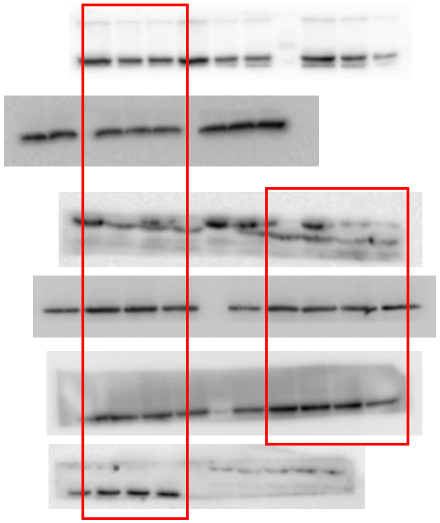

Figure 6H

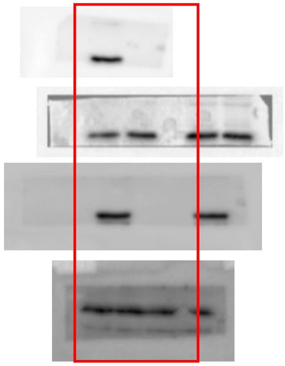

Figure 6I

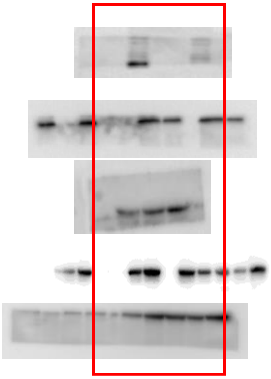

Figure 6J

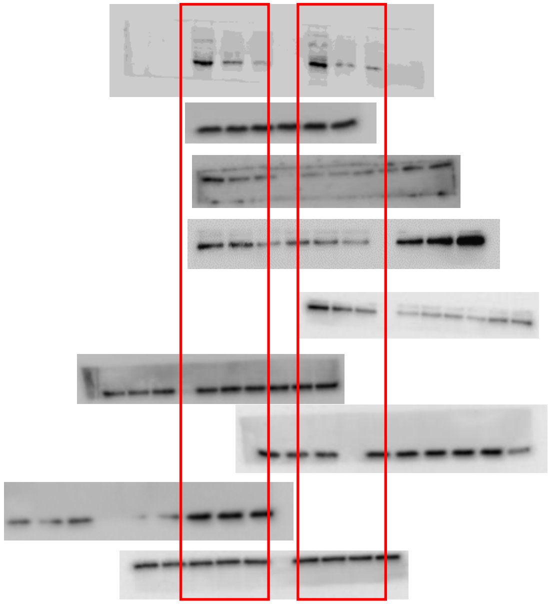

Figure 6K

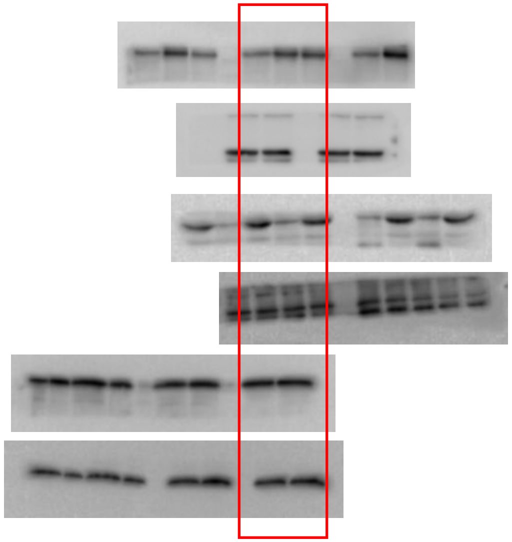

Figure 6L

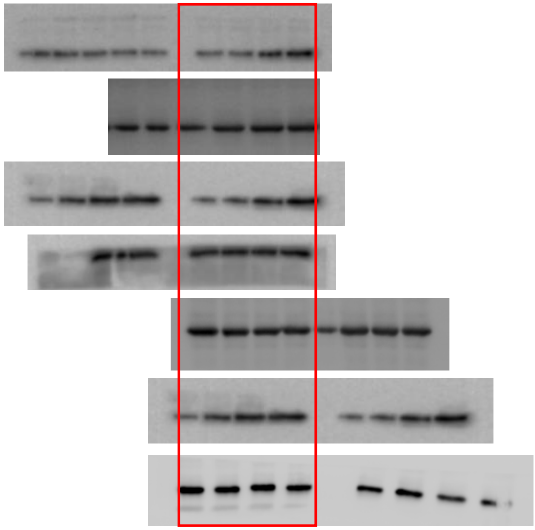

Figure 7D

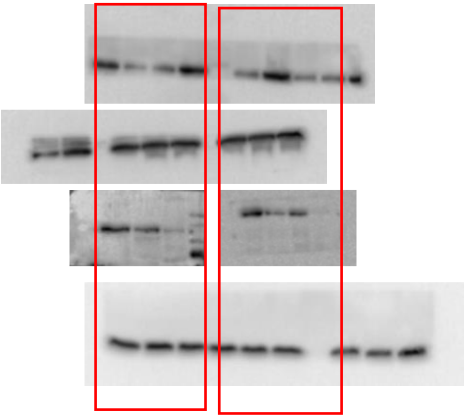

Figure 7E

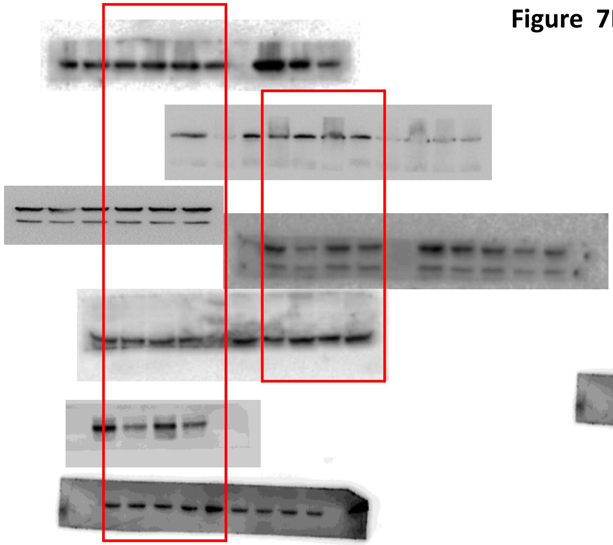

Figure 7F

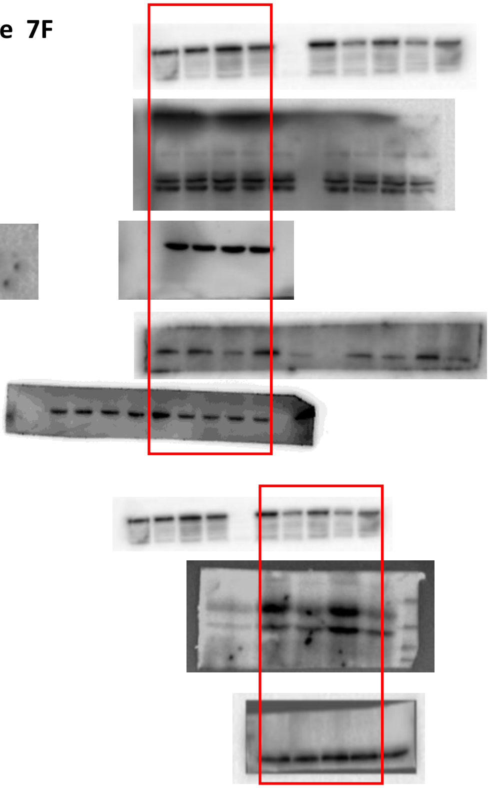

Figure 7H

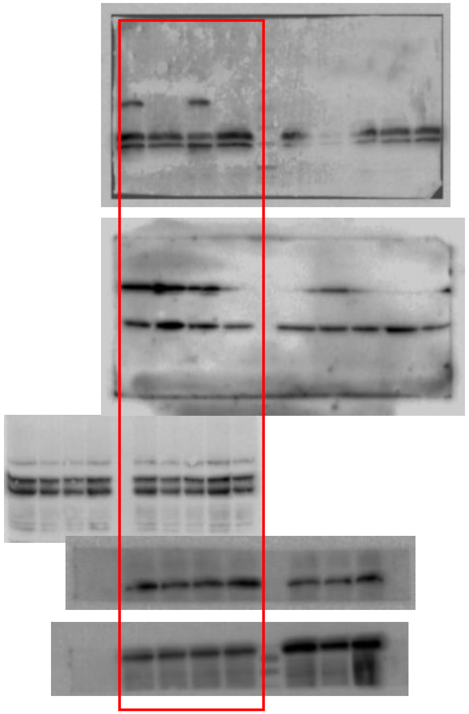

Figure 7J

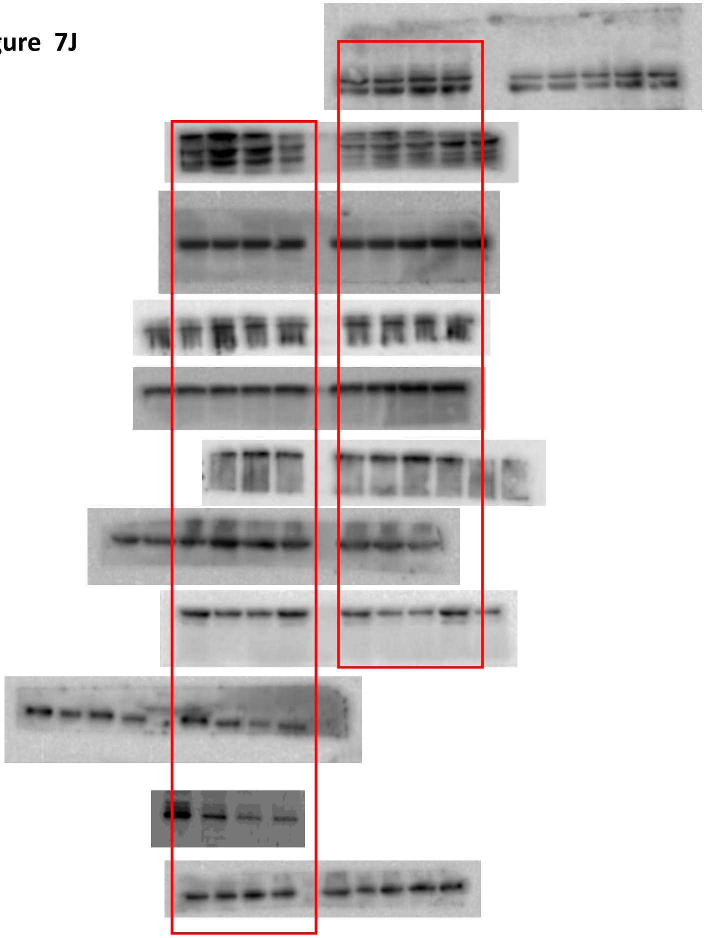

Figure S1C

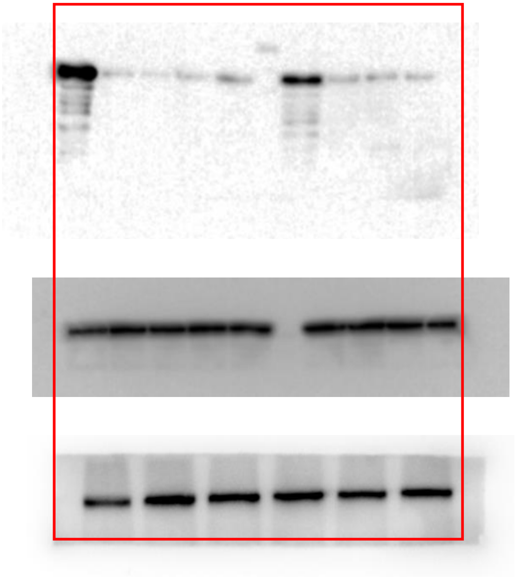

Figure S1H

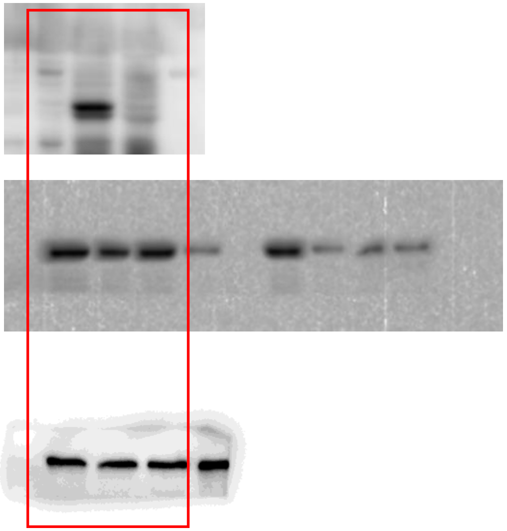

Figure S2A

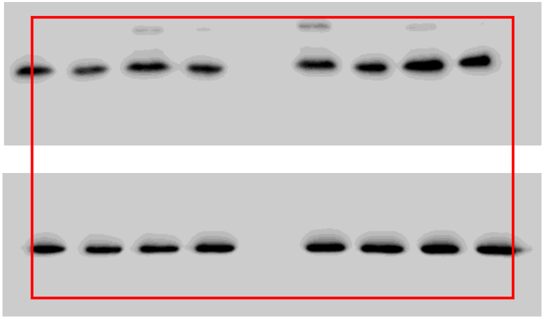

Figure S2B

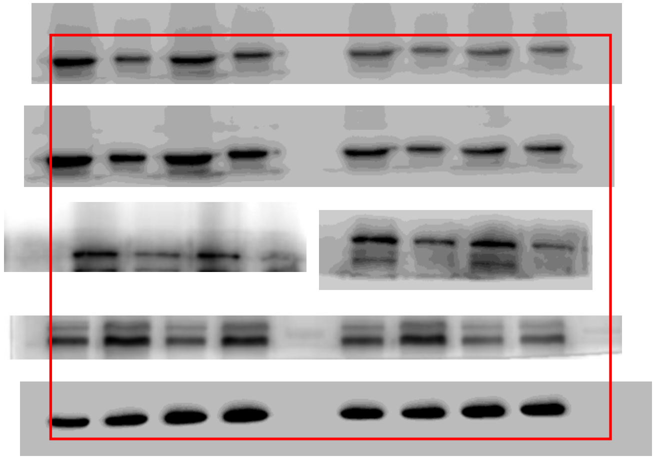

Figure S3B

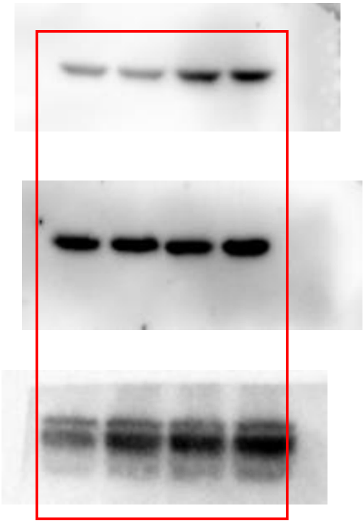

Figure S3C

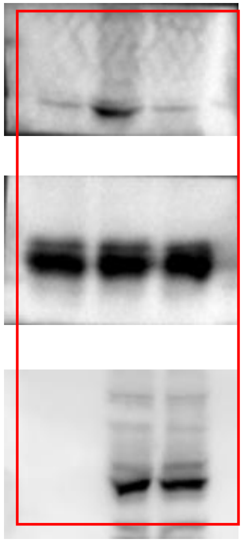

Figure S3D

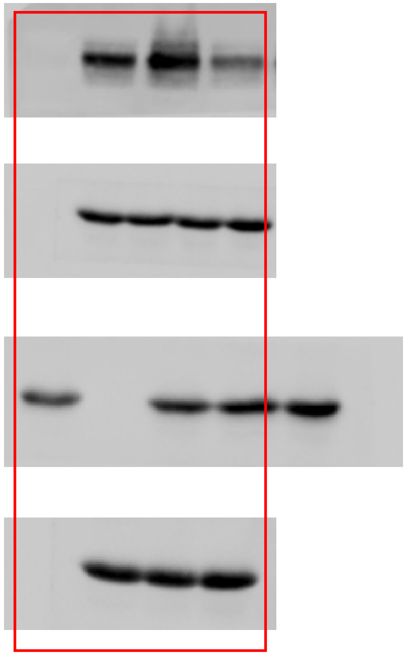

Figure S4A

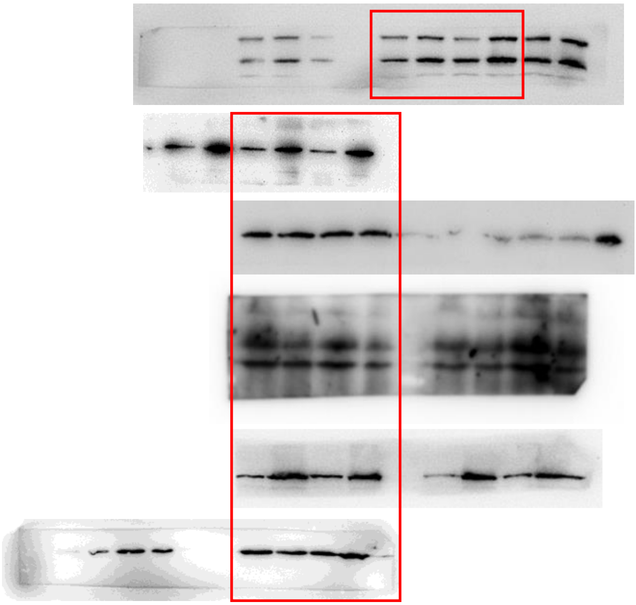

Figure S4C

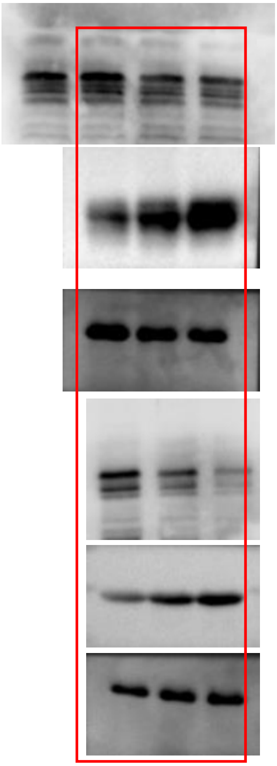

Figure S4D

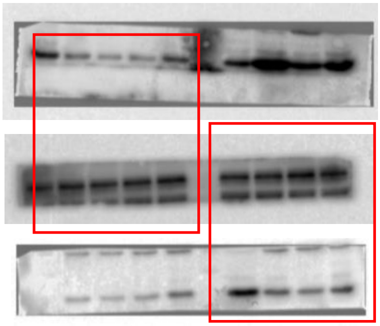

Figure S4E

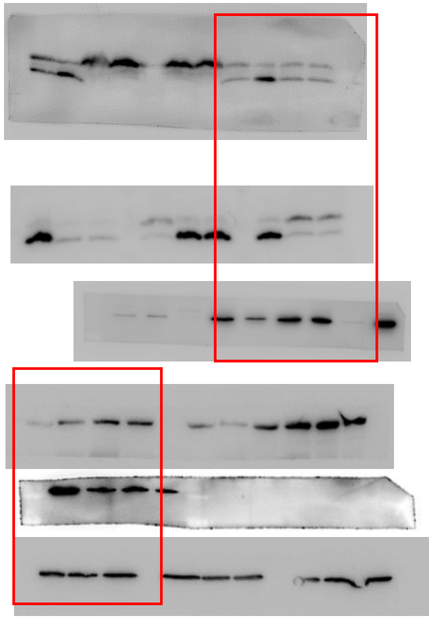

Figure S4G

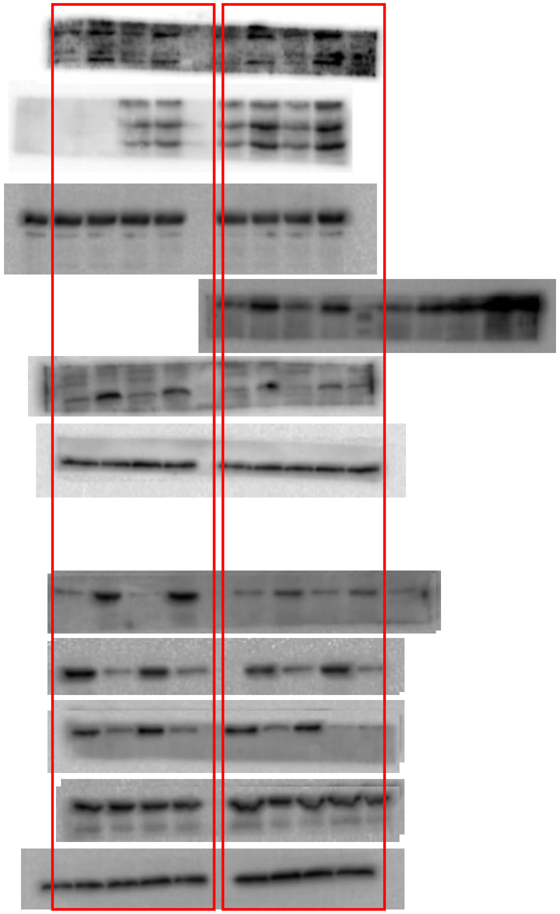

Supplement: Supplementary file 2 — Full Western Blots [file 41419_2026_8767_MOESM2_ESM.pdf]
