# Supplementary material for: Micropeptide SCAPEP triggers lung adenocarcinoma tumorigenesis via regulating autophagy by promoting CDK15-mediated phosphorylation of vimentin
Source: Cell Death Dis. 2026 May 6;17(1):602. doi: 10.1038/s41419-026-08767-1 (PMC13315923; doi:10.1038/s41419-026-08767-1)
Supplement: Supplementary file 3 — Supplementary Tables 1-5 [file 41419_2026_8767_MOESM3_ESM.docx]

**Supplementary Table S1.** Correlation of the expression of SCAPEP with clinicopathologic features in non-small cell lung cancer.

| Variables | N (%) | SCAPEP ^a^ | | p-value |
| --- | --- | --- | --- | --- |
|  |  | High | Low |  |
| Gender  Male  Female |  |  |  | 0.1735 |
|  | 48(53%) | 24 | 24 |  |
|  | 42(47%) | 28 | 14 |  |
| Age(years)  <55  ≥55 |  |  |  | 0.0501 |
|  | 27(43%) | 11 | 16 |  |
|  | 63(57%) | 41 | 22 |  |
| Tumor size(cm)  ≥5  <5 |  |  |  | 0.0384* |
|  | 39(43%) | 16 | 23 |  |
|  | 51(57%) | 36 | 15 |  |
| Lymph node metastasis  NO  Yes |  |  |  | 0.0203* |
|  | 46(51%) | 19 | 27 |  |
|  | 44(49%) | 33 | 11 |  |
| TNM stage  Ⅰ+Ⅱ  Ⅲ+Ⅳ |  |  |  | 0.0326* |
|  | 38(42%) | 13 | 25 |  |
|  | 52(48%) | 39 | 13 |  |
| Tumor differentiation  High  Low |  |  |  | 0.0528 |
|  | 43(47%) | 20 | 23 |  |
|  | 47(43%) | 32 | 15 |  |

^a^ Fold change (FC) (tumor tissues relative to normal tissues) is greater than or equal to 2.0 for high expression, and less than 2.0 for low expression.

*P<0.05 was considered significant (Chi-square test between 2 groups)

**Supplementary Table S2.** The RT-PCR primers used in this study

| Gene names | Primers Sequences |
| --- | --- |
| hsa_circ_0065214 | Forward: AGGAGATTGGTGTCGCTGAG |
| Divergent | Reverse: AGTGGGTAGCCGCCATTG |
| hsa_circ_0065214  Convergent  SCAP  hsa_circ_0003028  hsa_circ_0084443  hsa_circ_0011385  Vimentin  CDK15  MTRR  U6  GAPDH | Forward: ACTTCTGGCAGAATGACTGGGA  Reverse: TACCATGCCAAGTTCCTGGGCA  Forward: CAGTGCTGTCAAGTGTGTGC  Reverse: AGCCCATGGTTGTAGAAGGC  Forward: GTCCAAGATTCTGGCAAAGC  Reverse: TCAAAGAGATCCTCCTGGTGA  Forward: GCCTGGATGGATACCTGAAG  Reverse: GATGAGGGGCAACCTTGTAG  Forward: GACAACAATGAGCCCTACA  Reverse: AGCCGAATTGGTCTTGAGAA  Forward: GGACCAGCTAACCAACGACA  Reverse: AAGGTCAAGACGTGCCAGAG  Forward: CTTCAACTCGCCACAGGCTA  Reverse: CTTCAACTCGCCACAGGCTA  Forward: CCACCAGCCTCTCCTTGAATA  Reverse: GGCTGAGTCGTGATTTTCGG  Forward: CTCGCTTCGGCAGCACA  Reverse: AACGCTTCACGAATTTGCGT  Forward: CTCCTCCTGTTCGACAGTCAGC  Reverse: CCCAATACGACCAAATCCGTT |

**Supplementary Table S3.** The shRNAs and siRNAs used in this study

| siRNA | Sequences |
| --- | --- |
| sh-circ_0065214-1  sh-circ_0065214-2 | CUCAAUGGCGGCUACCCACATT  CACCCUCAAUGGCGGCUACATT |
| si-Vimentin-1  si-Vimentin-2  si-CDK15-1  si-CDK15-2  si-MTRR-1  si-MTRR-2 | UCGAGGUGGAGCGCGACAAATT  CUACAUCGACAAGGUGCGCATT  GAGCUGGTGCUUACAGAGAATT  UCUGUCCAGGGAUCAUCUCATT  AGCCCUGGCUCCUAAGAUAATT  AGGCAUAAGGAUAGGGAUUATT |

**Supplementary Table S4**. Antibodies used in this study

| Antigens | Manufacturers | Applications | dilution |
| --- | --- | --- | --- |
| β-actin mAb | A3854, Sigma-Aldrich | WB | 1:5000 |
| SCAPEP mAb  vimentin pAb  CDK15 mAb  P62 pAb  LC3 pAb  Smad2/3 pAb  P-Smad2 pAb  H3 pAb  MTRR pAb  ERK1/2 pAb  AKT mAb  P-AKT pAb  mTOR  p-mTOR  p-Ser/Thr  DDDDK-tag pAb  DYKDDDDK-tag mAb  HA-tag mAb  His-tag mAb  ki-67(V3242) pAb  HRP-linked anti-rabbit IgG  HRP-linked anti-Mouse IgG  Goat Anti-Rabbit IgG(H+L) Cy3  Goat Anti-Mouse IgG(H+L) 488 | ABclonal  10366-1-AP, proteintech  TA811952,  Thermo Fisher Scientific  18420-1-AP, proteintech  14600-1-AP, proteintech  A1933, ABclonal  A19544, ABclonal  17168-1-AP, proteintech  26944-1-AP, proteintech  28733-1-AP, proteintech  60203-2-Ig, proteintech  28731-1-AP, proteintech  66888-1-Ig, proteintech  67778-1-Ig, proteintech  ab17464, Abcam  AP0007, Biogot Technology  MA1-91878,  Thermo Fisher Scientific  AP0005M, Biogot Technology  BS67270, Biogot Technology  BS1454, Biogot Technology  BS13278, Biogot Technology  BS12478, Biogot Technology  BS10007, Biogot Technology  BS10015, Biogot Technology | WB/IF/IHC  WB/IP/IF  WB/IP/IF  WB  WB  WB/CHIP  WB  WB  WB  WB  WB  WB  WB  WB  WB  WB  IP/IF  WB/IP  WB/IP  IHC  WB  WB  IF  IF | 1:500  1:1000  1:1000/  1:200  1:2000  1:1000  1:2000  1:2000  1:3000  1:1000  1:2000  1:1000  1:1000  1:1000  1:1000  1:1000  1:2000  1:2000/  1:500  1:2000  1:2000  1:500  1:5000  1:5000  1:1000  1:1000 |

**Supplementary Table S5.** The top 25 proteins that specifically interact with SCAPEP were identified by mass spectrometry.

| UniProt ID | Gene names | Sequence coverage (%) | | Unique Peptides | Score |
| --- | --- | --- | --- | --- | --- |
| P08670 | Vimentin | | 43.6 | 20 | 210.19 |
| P63261 Q15149  Q5TCU3 P02461  O95239 P35579  A0A0C4DGC5 A0A494C0Y7  F8WDP7  A0A7I2V3Q4 E5RIH6  F8W9F8 H3BM14  A0A7I2V5U9 Q5VW31  A0A2R8Y5P9 A0A2R8YG28  A6XGL3 E7ERT8  H0Y420 P20930  P35030 Q5T2N8  Q5T750 | ACTG1  PLEC  TPM2  COL3A1  KIF4A  MYH9  LMNA  RAD50  CDK15  HSP90AB1  CEP170  A1CF  NUB1  HNRNPA1  NFIB  SHROOM3  OTOA  PRSS1  HOXA1  COL17A1  FLG  PRSS3  ATAD3C  XP32 | | 34.1  1  9.2  2.7  1.1  0.8  3.1  1.2  13  1.5  6.8  1  1  12  6  0.5  1.7  3.4  4.4  22.7  0.2  4.3  1.7  3.2 | 12  4  3  3  3  2  2  2  1  1  1  1  1  1  1  1  1  1  1  1  1  1  1  1 | 115.96 24.717  18.997 19.152  11.517 33.694  7.011 6.1147  6.0789  10.003 6.0772  9.6999 6.3957  7.7652 6.4382  6.173 6.5008  6.2183 6.6266  7.8349 6.2606  6.0487 7.3947 6.69 |
